# Characterization of Pro-Inflammatory Flagellin Proteins Produced by *Lactobacillus ruminis* and Related Motile Lactobacilli

**DOI:** 10.1371/journal.pone.0040592

**Published:** 2012-07-10

**Authors:** B. Anne Neville, Brian M. Forde, Marcus J. Claesson, Trevor Darby, Avril Coghlan, Kenneth Nally, R. Paul Ross, Paul W. O’Toole

**Affiliations:** 1 Department of Microbiology, University College Cork, Cork, Ireland; 2 Alimentary Pharmabiotic Centre, University College Cork, Cork, Ireland; 3 Teagasc, Moorepark Food Research Centre, Moorepark, Fermoy, Co. Cork, Ireland; Columbia University, United States of America

## Abstract

*Lactobacillus ruminis* is one of at least twelve motile but poorly characterized species found in the genus *Lactobacillus*. Of these, only *L. ruminis* has been isolated from mammals, and this species may be considered as an autochthonous member of the gastrointestinal microbiota of humans, pigs and cows. Nine *L. ruminis* strains were investigated here to elucidate the biochemistry and genetics of *Lactobacillus* motility. Six strains isolated from humans were non-motile while three bovine isolates were motile. A complete set of flagellum biogenesis genes was annotated in the sequenced genomes of two strains, ATCC25644 (human isolate) and ATCC27782 (bovine isolate), but only the latter strain produced flagella. Comparison of the *L. ruminis* and *L. mali* DSM20444^T^ motility loci showed that their genetic content and gene-order were broadly similar, although the *L. mali* motility locus was interrupted by an 11.8 Kb region encoding rhamnose utilization genes that is absent from the *L. ruminis* motility locus. Phylogenetic analysis of 39 motile bacteria indicated that *Lactobacillus* motility genes were most closely related to those of motile carnobacteria and enterococci. Transcriptome analysis revealed that motility genes were transcribed at a significantly higher level in motile *L. ruminis* ATCC27782 than in non-motile ATCC25644. Flagellin proteins were isolated from *L. ruminis* ATCC27782 and from three other *Lactobacillus* species, while recombinant flagellin of aflagellate *L. ruminis* ATCC25644 was expressed and purified from *E. coli*. These native and recombinant *Lactobacillus* flagellins, and also flagellate *L. ruminis* cells, triggered interleukin-8 production in cultured human intestinal epithelial cells in a manner suppressed by short interfering RNA directed against Toll-Like Receptor 5. This study provides genetic, transcriptomic, phylogenetic and immunological insights into the trait of flagellum-mediated motility in the lactobacilli.

## Introduction

Bacterial flagella are long, thin, proteinaceous structures that form rigid helices which rotate in a counterclockwise direction to propel the cell forward [1], [2]. The filament of the bacterial flagellum is composed of polymers of a protein called flagellin, a microbe associated molecular pattern (MAMP) that is recognized by toll-like receptor 5 (TLR5) of the host [3], and which activates the nuclear factor-κβ (NFκβ) signaling-pathway in epithelial and innate immune cells [4]–[6]. While lactobacilli have been researched intensively because of their food and health-related applications [7], to date, neither their flagella nor their capacity for flagellum-mediated motility has been formally characterized.

From a microbial perspective, flagellate species may have a competitive advantage over non-motile species with respect to niche colonisation [8], biofilm formation [9], [10] and for the secretion of virulence proteins by pathogens [11]. However, the production of flagella requires a substantial investment of resources by the bacterium, with ∼0.1% and ∼2% of the cell’s energy devoted to flagellum rotation and biosynthesis respectively [12]. For this reason, and also presumably to avoid stimulating immune responses, some species, such as *Listeria monocytogenes* suppress motility gene expression *in vivo* [13], [14].

Recently, the crystal structures of human TLR5 (hTLR5) [15] and also of *Danio rerio* (zebrafish) TLR5 (drTLR5) in complex with two *Salmonella* flagellin molecules [16] have been determined, providing insight into the molecular-basis for flagellin recognition by TLR5. In the absence of its ligand, hTLR5 exists as an asymmetric homodimer [15] and like the drTLR5, this structure is proposed to bind two flagellin molecules [15]. Previously, alanine scanning mutagenesis had identified specific flagellin residues that reduced TLR5 recognition of flagellin by 76–97% when mutated [17]. Several of these critical residues were found on the convex surface of the flagellin molecule and include core flagellin residues R90, L94 and Q97 [17]. The structural studies confirmed that R90, L94 and Q97 were indeed involved in the TLR5 interaction, and bonded with several TLR5 residues at the primary binding interface B of the drTLR5 structure [16]. In particular, the LRR9 loop of drTLR5 formed the major flagellin binding site [16]. Specifically, flagellin residues R90 and E114 have been shown to interact with several drTLR5 residues, including TLR5 S271 [16], a naturally varying TLR5 residue that is involved in establishing the flagellin recognition profile of a given species [18].

Several flagellate bacterial pathogens have evolved flagellin proteins that are not recognized by human TLR5 [19]. Residues 89–96 of the amino-terminal D1 domain of *S. typhimurium* flagellin constitute the flagellin pattern recognized by TLR5. These residues are also involved in flagellin polymerization [17], [19]. Particular substitutions within this region enable selected flagellate α- and ε-proteobacterial pathogens, including *H. pylori* and *C. jejuni*, to evade immune-recognition without compromising their motility [19]. While the recognition of *Lactobacillus* cells and their associated molecules by TLRs 2, 4 and 9 has been well established [20], [21] a direct TLR5-*Lactobacillus* interaction has not been demonstrated previously.

At present, at least twelve motile species have been officially recognized in the genus *Lactobacillus* (Table S1), and the *L. salivarius* phylogenetic clade [22] includes the largest proportion of these motile species. Eleven of these twelve motile *Lactobacillus* species have been isolated from food or environmental sources (Table S1). *L. curvatus* subsp. *curvatus* and *L. sakei* subsp. *carnosus* which were described as motile in one study [23], are located in the *L. sakei* clade.

Lactobacilli represent a subdominant element of the human intestinal microbiota. By culture-dependent methods, the faecal *Lactobacillus* population of adult humans was estimated at approximately 10^4^–10^8^ CFU/g faeces (wet weight) [24]–[26]. Culture-independent enumeration approaches, such as real-time PCR [27], [28] similarly suggest that the lactobacilli are present in the faecal microbiota at concentrations of 10^6^–10^8^ targets/g faeces (wet weight). Therefore, the lactobacilli represent at most ∼0.01%–0.6% of the faecal microbiota, and this proportion varies significantly from individual to individual [29], [30].

Certain *Lactobacillus* species and strains have been developed as probiotics for human consumption [31]. Accordingly, the potential *in vivo* functions of cell-surface appendages of lactobacilli, such as pili [32] and surface layer proteins [33], [34] have been investigated. The flagellate *Lactobacillus* species however, have not attracted much scientific attention thus far, and the perception that the lactobacilli are uniformly non-motile persists as a consequence [35], [36]. Moreover, well characterized, aflagellate *Lactobacillus* species have been engineered to display *Salmonella* flagellin proteins on their cell-surface with the intention of developing these recombinant strains as vaccine delivery vectors with enhanced adjuvancy [37], [38]. Naturally flagellate *Lactobacillus* species have been overlooked for this purpose to date.

*L. ruminis* has been identified as part of the intestinal microbiota of several mammals, including humans [29], [39]–[41], cows [42], [43] and pigs [44], [45], and it is the only formally recognized motile *Lactobacillus* species which is also autochthonous to the gastrointestinal tract of humans [29], [41]. *L. ruminis* is an obligately homofermentative rod-shaped bacterium that tends to form end-to-end filaments or chains of cells [46]. Bovine *L. ruminis* isolates, including strain ATCC27782 which is one of the strains analyzed in this study, are motile by means of peritrichous flagella [42]. Although the first report of *L. ruminis* (then called *Catenabacterium catenaforme*) isolation from humans described the bacterium as being motile [47], the strain lodged, (later identified as *L. ruminis* ATCC25644) was non-motile [41].

*Lactobacillus mali* [48] is one of the twelve motile species found in the twenty-five- member *L. salivarius* clade. Historically, the formal description of this species has been complicated by several revisions of its nomenclature [49], [50]. In general, the scientific literature describing the biology of *L. mali* is sparse. Nevertheless, its status as a wine and cider associated lactic acid bacterium has yielded some insight into the metabolism of the species. *L. mali* is a malolactic bacterium [51] capable of producing volatile phenols [52], [53] and biogenic amines [54], [55] which influence the organoleptic properties and the safety of fermented alcoholic beverages. *L. mali* strains are also known to produce exopolysaccharide [56] and menaquinones [46], [57] suggesting that some *L. mali* strains may be suitable for industrial applications.

We have determined the genomic basis for flagellum-mediated motility in the genus *Lactobacillus* by investigating the motility loci of two *L. ruminis* strains and comparing them to the motility locus of *L. mali*. We have considered the phylogenetic origins of the *L. ruminis* motility genes among the motile species of the order *Lactobacillales*. We have also investigated the non-motility of *L. ruminis* isolates of human origin, and we have characterized the flagellin proteins of, and the innate immune response to, these motile and non-motile autochthonous gastrointestinal (GI) commensals.

## Results

### Motility of Lactobacilli

**Figure 1:**
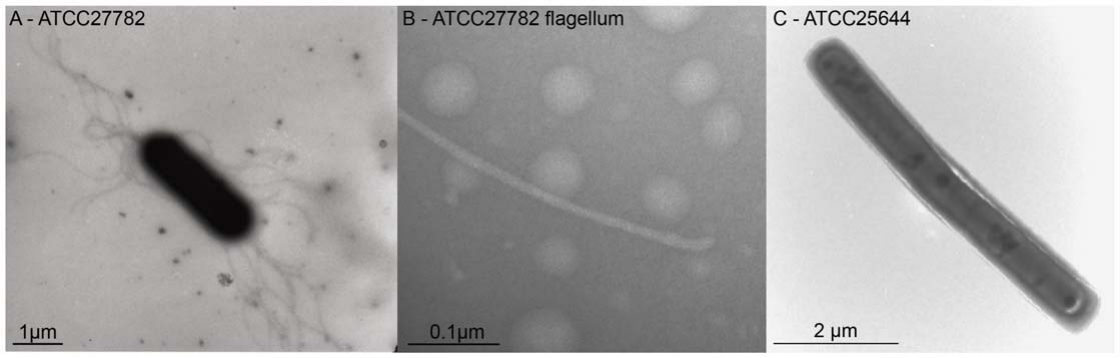
Transmission electron micrographs of *L. ruminis* whole cells and flagella. A: *L. ruminis* ATCC27782; 1% ammonium molybdate, 6000× magnification. B: *L. ruminis* ATCC27782 flagellum filament with visible attached hook structure; 2% ammonium molybdate, 250000× magnification. C: *L. ruminis* ATCC25644; 0.25% ammonium molybdate, 15000× magnification.

Of nine *L. ruminis* strains examined, only strains isolated from cows, (ATCC27780, ATCC27781 and ATCC27782) were flagellate and motile (Figure 1, Table S2). These strains displayed unicellular motility, but also often formed chains of two to five cells which were motile as a unit (Movie S1). The observed motility of *L. ruminis* ATCC27782 typically decreased with increasing cell density during growth. During early exponential phase, cultures were often “very motile” to “moderately motile” when viewed with a phase contrast microscope. At late exponential phase, cultures became “weakly motile”, while stationary phase cultures were typically non-motile. The motility of stationary phase cells could be restored by adding fresh medium, suggesting that nutrient depletion may influence the motility phenotype. Three other species of the *L. salivarius* clade tested, specifically *L. ghanensis* L489^T^, *L. mali* DSM20444^T^ and *L. nagelii* DSM13675^T^ were also motile. *L. ruminis* isolates from humans, including ATCC25644, were not motile when grown in MRS media as determined by phase-contrast microscopy and motility agar plates.

### Annotation of *L. ruminis* and *L. mali* Motility Genes

“High-quality draft” and “finished” [58] genome sequences were generated for *L. ruminis* ATCC25644 and ATCC27782 respectively [59]. Motility-related genes were annotated in loci spanning 45,687 bp (ATCC25644) and 48,062 bp (ATCC27782). The [GC] % of these genomic regions was 44.79% (ATCC25644) and 44.04% (ATCC27782), consistent with the overall values for each genome of 43.76% and 43.34% respectively.

A “standard draft” genome 2.6 Mb in size, consisting of 200 contigs assembled into 95 scaffolds, was generated for *L. mali* DSM20444^T^. Its motility locus spans 51, 309 bp, and it encodes forty three motility and chemotaxis genes. Its [GC]% of 36.2% is consistent with the [GC]% of 36% for the whole genome.

### Comparative Genomics and Phylogeny of *Lactobacillus* Motility Genes

The motility loci of *L. ruminis* ATCC25644 and ATCC27782 both encode forty-five predicted flagellum biogenesis and chemotaxis genes (Figure S1; Table S3), but with a second copy of the *fliC* gene (*fliC2*) and a glycosyltransferase pseudogene being additionally present in the ATCC27782 genome. These motility loci otherwise display conserved order and orientation in the two genomes and are 97% identical to each other at nucleotide level.

The *L. mali* genome encodes a similar set of motility genes, and its genetic arrangement is broadly similar to that of the *L. ruminis* ATCC27782 motility locus (Figure S2). The *L. mali* motility genes are divided between two loci that are separated between *flgL* and *fliC*, by an ∼11.8 Kb region that encodes a number of metabolic genes, including those for rhamnose metabolism (*rhaTBMAD*). Relative to the *L. ruminis* motility locus, other notable variations in the *L. mali* motility region include the inversion of the *motAB* gene pair, the absence of homologs for *flaG* and a potential negative regulator (LRC_15730/ANHS_518; see below) of motility gene expression and the presence of only one flagellin gene in a strain that is known to be motile.

The relatedness of the *L. ruminis* motility proteins to those of 39 other *Firmicutes*, *Proteobacteria* and *Thermotogae* was evaluated by generation of phylogenetic trees for 39 motility protein families. A well supported *L. ruminis*-*Carnobacterium*-*Enterococcus* clade was present in 22 motility protein trees and in the 16S rRNA gene tree (Figure S3). These data suggest that the *L. ruminis* motility genes were probably acquired by vertical descent via the *L. ruminis-Carnobacterium-Enterococcus* last common ancestor.

### Proposed Regulation of *L. ruminis* Motility Gene Transcription and Bioinformatic Analysis of ANHS_518/LRC_15730

The transcription of motility genes in *L. ruminis* ATCC25644 and ATCC27782 was examined by microarray analysis during the motile and non-motile growth phases, to investigate the genetic basis for their different motility phenotypes. During the motile phase of growth, transcription of most genes in the motility locus was significantly higher in ATCC27782 than in ATCC25644 (Table S4), with the exception of LRC_15730, a gene on the opposite DNA strand (Figure S1). This gene was transcribed at a much higher level in ATCC25644 (annotated as ANHS_518 in ATCC25644 (Table S4)), suggesting that it may function as a negative regulator of flagellum biogenesis. Microarray data also showed that flagellin genes (*fliC1* and *fliC2*) were transcribed at much higher levels in ATCC27782 than in ATCC25644. Since the *fliC1* and *fliC2* genes are 98% identical and could not be distinguished by their hybridization to the array oligonucleotides, allele-specific qRT-PCR was used to show that only *fliC2* was transcribed at high levels in ATCC27782, with Cq values of ∼23, compared to Cq values of ∼37 (indicating very low expression) and 0 (non-expression) for *fliC1* in ATCC27782 and ATCC25644 respectively. Thus *fliC2* is the majorly expressed flagellin gene in the motile strain, and *fliC1* may be expressed at very low levels only in ATCC27782. The *era* gene comparator had a Cq value in the range of 18–19.5. Microarray data was also verified by qRT-PCR for *fliM* and LRC_15730/ANHS_518.

Four sigma factors, σ^70^, σ^54^, σ^28^, and σ^70′^(a σ^70^-like putative extracytoplasmic function sigma factor) were annotated in the *L. ruminis* genome [59]. These genes are shown schematically in Figure S4). Relative transcription of σ^28^ (the flagellin-specific *fliA* sigma factor) was significantly higher in ATCC27782 than in ATCC25644. In contrast, the σ^70′^ gene was expressed at much higher level in ATCC25644 than in ATCC27782. The genes for the housekeeping sigma factors σ^70^ and σ^54^ were not differentially expressed between strains. Likewise there was no differential expression of the gene LRC_15720 which is part of the motility locus and may encode a sigma factor, but which is not formally annotated as such.

The transcription start (+1) sites of several candidate regulatory and effector genes involved in flagellum biogenesis were mapped by 5′ rapid amplification of cDNA ends (RACE) to assist promoter identification *in silico*. Both flagellin genes *fliC*1 and *fliC*2 of *L. ruminis* ATCC27782 appear to be under the control of a σ^28^-dependent promoter (Figure S4). The *fliC*1 gene transcription +1 site could not be experimentally determined because this gene is not highly expressed in either ATCC25644 or ATCC27782. Nevertheless, the sequence identity and spacing of the predicted −35 and −10 boxes at the promoters of *fliC*1 and *fliC*2 were broadly conserved (Figure S4). Thus, *fliC*1 is predicted to share the same promoter configuration and type as that of *fliC*2. A short palindromic sequence AGATCT, a known transcription factor binding site [60], and the recognition sequence of the restriction enzyme BglII, was identified between the −35 and −10 elements of the *fliC*1 in both *L. ruminis* ATCC27782 and ATCC25644. This palindrome was not present at the ATCC27782 *fliC*2 promoter.

Consistent with other *Lactobacillus* genes and transcripts [61], a purine residue (G) appears in the +1 position of the ANHS_518 gene transcript. However, the promoter region for this gene did not conform to the consensus sequences or motifs predicted for known *Lactobacillus* promoters [61] and so could not be reliably identified *in silico*.

Bioinformatic analyses revealed that the LRC_15730/ANHS_518 gene is unique to *L. ruminis* strains, meaning that it has no homologs in any other sequenced species. Iterative PSI-BLASTp searches revealed weak homology to a *B. cereus* ComK transcription factor (ZP_03237802.1, E-value 0.002) after seven iterations.

### Characterization of Flagellin Proteins Extracted from Motile *Lactobacillus* Species

**Figure 2:**
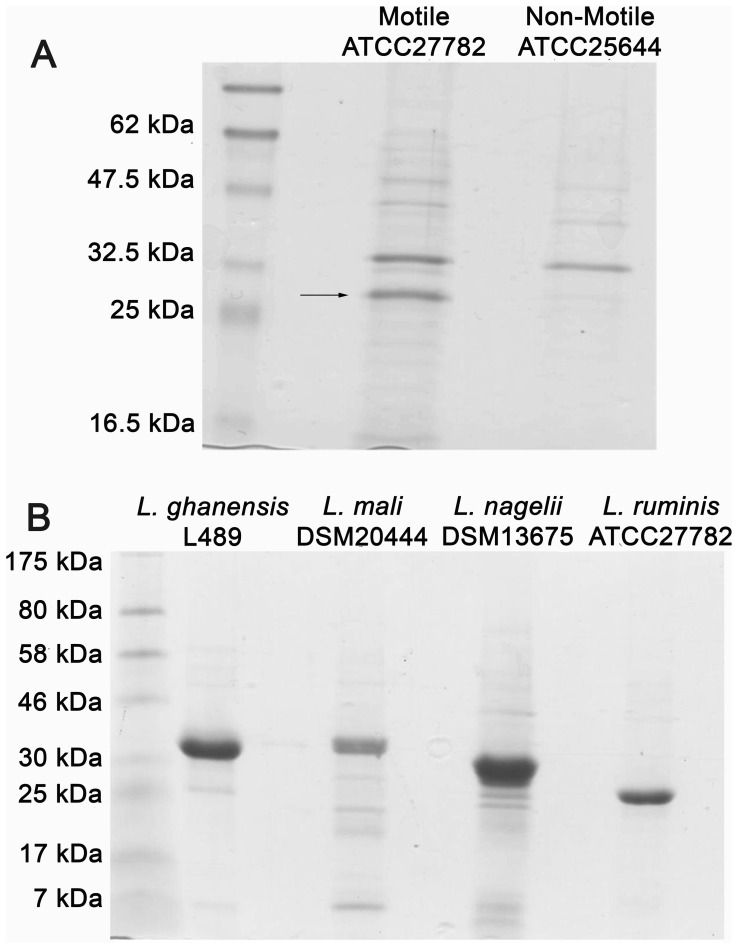
Extraction of *Lactobacillus* flagellin proteins and analysis on 10% SDS-PAGE gels. A: Pilot extraction of surface proteins from motile and non-motile *L. ruminis* strains. The arrow points to the ATCC27782 flagellin protein. The larger protein at ∼34 kDa is GAPDH. B: Flagellin proteins extracted from various *Lactobacillus* species as indicated.

Flagellin proteins were extracted from *L. ruminis* ATCC27782, *L. ghanensis* L489^T^, *L. mali* DSM20444^T^ and *L. nagelii* DSM13675^T^ (Figure 2). These candidate flagellin proteins ranged in size from ∼25 kDa to ∼38 kDa (Figure 2). Flagellin was not recovered when *L. ruminis* ATCC25644 (Figure 2) cells were subjected to the same extraction procedure. Fifteen amino-terminal residues of the *L. ruminis* ATCC27782 ∼26 kDa protein product were sequenced, while 10 residues of the other isolated flagellins were sequenced. All sequences were identical to the amino-terminus of the major *L. ruminis* flagellin proteins except for a Val7Ile substitution in *L. mali* and an Ala8Ser substitution in the *L. ghanensis*, *L. mali* and *L. nagelii* proteins. The chemically determined *L. ruminis* ATCC27782 flagellin protein sequence corresponded exactly to that predicted from the highly expressed *fliC2* gene from *L. ruminis* ATCC27782, but also to the predicted (but not expressed) *fliC1* gene products of both strains. The ∼34 kDa protein present in both the ATCC27782 and ATCC25644 protein samples (Figure 2, panel A) was also amino-terminally sequenced. It was identified as glyceraldehyde 3-phosphate dehydrogenase (GAPDH), which is commonly found on the surface of Gram–positive bacteria [62].

### Flagellate *Lactobacillus* Cells, Native and Recombinant *Lactobacillus* Flagellin Proteins Stimulate IL8 Secretion from Human Intestinal Epithelial Cell Lines

**Figure 3:**
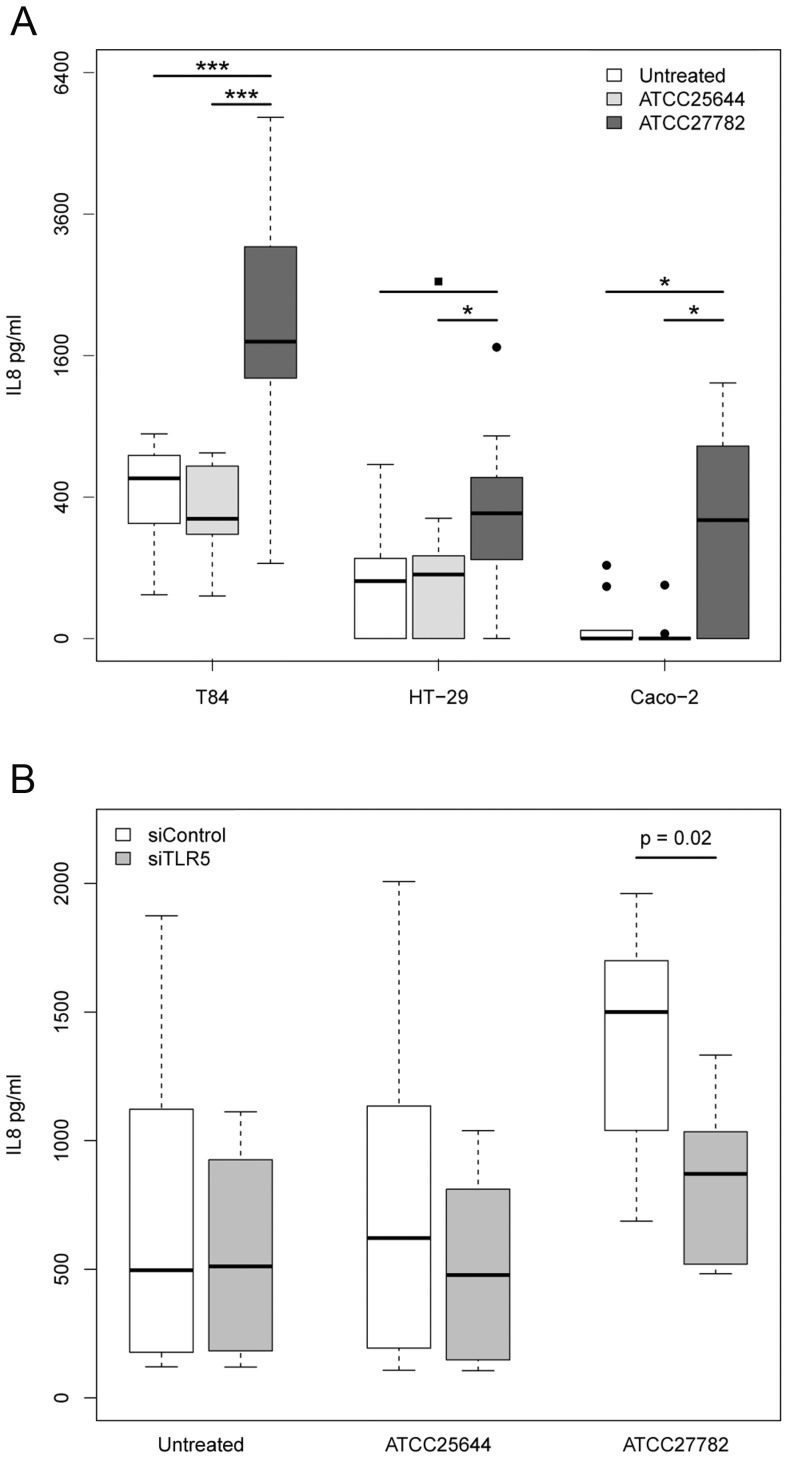
Flagellate *L. ruminis* cells or *Lactobacillus* flagellin stimulate IL8 production by intestinal epithelial cells in a TLR5-dependent manner. A: Flagellate *L. ruminis* ATCC27782 cells induce significant IL8 secretion from human intestinal epithelial cell lines. T84, n = 9; HT-29, n = 7; Caco-2, n = 6. The increments on the Y-axis follow a square-root scale. B: Following transfection with siRNA directed against TLR5, IL8 secretion from HT-29 cells was significantly lower in response to ATCC27782 whole bacteria. Boxplots are based on six experimental replicates. Box-plots show the median and the interquartile range. Solid black dots represent outlier values. Statistical significance was determined using a one-tailed Mann-Whitney U test. Horizontal black bars indicate significant differences between samples. The level of statistical significance is given by P-values over these bars, or by the following symbols: P<0.001 = ***; P<0.05 = *; P<0.1 = ▪.

Interleukin 8 (IL8) production is a central component of the inflammatory response to bacterial flagellin proteins [5]. Flagellate *L. ruminis* ATCC27782 cells elicited significantly more IL8 secretion from three human colonic epithelial cell lines (T84; HT-29, Caco-2), than the aflagellate ATCC25644 strain (Figure 3) (P≤0.01). In fact, ATCC25644 did not induce significant IL8 secretion from any of the cell lines tested. Furthermore, the flagellin proteins of *L. ghanensis*, *L. mali* and *L. nagelii* also induced significant IL8 secretion from HT-29 cells when compared to the untreated control (P<0.01) (Figure S5). Taken together, these data suggested that the flagellin protein rather than another cell surface component, was responsible for much of the IL8 secretion. To demonstrate that the *Lactobacillus* flagellin-TLR5 interaction was indeed responsible for the IL8 secretion observed, HT-29 cells were transfected with siRNA targeting TLR5 to reduce the expression of this receptor. These cells were stimulated post-transfection with either *L. ruminis* ATCC25644 or ATCC27782 whole cells (Figure 3). Knockdown of TLR5 expression was confirmed by qRT-PCR and siRNA treatment did not impact on the viability of the epithelial cells (data not shown). IL8 secretion by HT-29 cells in response to whole flagellate *L. ruminis* ATCC27782 was significantly lower when TLR5 expression was reduced (P = 0.02) (Figure 3). Thus, *Lactobacillus* flagellin elicits IL8 secretion from intestinal epithelial cells in a TLR5 dependent manner.

A recombinant FliC-GST fusion protein was recovered from *E. coli* inclusion bodies to test if the non-expressed FliC1 protein of the aflagellate *L. ruminis* ATCC25644 strain would also elicit a proinflammatory response from T84 cells. Significantly more IL8 was secreted by T84 cells in response to the recombinant GST-flagellin fusion protein than in response to purified glutathione-S-transferase (GST) protein (P = 0.015), (Figure S5). The concentration of IL8 secreted by the T84 cells in response to the fusion protein ranged from 341.22–2065.64 pg/ml (n = 7) as determined by ELISA. This large range of IL8 secretion may be attributable to the nature of the fusion protein, which tended to lose its solubility and probably also its functional conformation upon concentration and cold-storage.

Several attempts were also made to express ATCC27782 *fliC1* recombinantly in *E. coli*. However, the *fliC1* and *fliC2* genes of ATCC27782 are 98% identical at a nucleotide level, and 98% identical at a protein level. As a result, during PCR to amplify the target gene for cloning purposes, chimeric molecules were formed. This meant that the amplified product would encode a flagellin protein which had some unique residues corresponding to *fliC1* and others corresponding to *fliC2*. For this reason, ATCC27782 *fliC1* was not expressed recombinantly.

### *L. ruminis* ATCC25644 May be Motile *in vivo*

To determine if *L. ruminis* ATCC25644 might regain motility *in vivo*, isogenic, rifampicin resistant ATCC25644 and ATCC27782 strains were individually fed to mice and were later recovered from their faeces. Following passage through the murine GI tract, a tumbling motility phenotype was imparted to some of the recovered *L. ruminis* ATCC25644 bacteria. Thus, some bacteria in these ATCC25644 cultures displayed tumbling, but not running motility when grown in MRS broth under standard conditions as assessed by phase-contrast microscopy (Movie S2). These bacteria were confirmed as ATCC25644 by strain specific PCR, 16S rRNA gene sequencing and carbohydrate profiling (data not shown).

To assess the impact of growth medium composition on the motility of ATCC25644, MRS motility agar plates were prepared from basic ingredients to which were added alternative carbon, protein and phosphate sources. None of these modifications imparted motility to ATCC25644 cultures as determined by phase-contrast light microscopy and semi-solid agar plates (Figure S6).

The motility of ATCC27782 was similarly evaluated in semi-solid agar in Falcon tubes with various supplements in a further attempt to simulate conditions in the gut that might promote motility. MRS agar supplemented with 1% porcine or bovine bile were tested which rendered the bacteria non-motile, while 0.5 mM EDTA, 1% NaCl and half-strength MRS did not abolish the motility of this strain as assessed by growth in semi-solid agar under anaerobic conditions at 37°C. None of these conditions induced motility in ATCC25644 in semi-solid agar (data not shown).

Altering the incubation temperature influenced the growth rate of ATCC27782, but did not impact on flagellin production. ATCC27782 that had been incubated in MRS broth at 25°C, 30°C, 37°C and 42°C achieved optical densities (600 nm) of ∼0.1, ∼0.2, ∼1.36 and ∼1.93 respectively after 16 hr incubation in MRS broth, (n = 3). Flagellin could be recovered from cultures that had been incubated at 30°C, 37°C and 42°C as demonstrated by Western blotting (data not shown). Motile bacteria were observed in the cultures incubated at 25°C, 30°C and 37°C after 16 hrs. Cultures grown at 42°C reached higher cell densities than those grown at lower temperatures, but lost motility at these high cell densities. Too few motile bacteria were present in the culture grown at 25°C to allow the recovery of flagellin.

## Discussion

As the number of *Lactobacillus* species described as being motile has been steadily increasing in recent years (Table S1), the biological significance of “motility” and of “flagella” in this genus merits investigation. From an evolutionary perspective, the almost complete restriction of flagellate species to the *L. salivarius* clade is also noteworthy.

“Motility” could potentially confer a number of competitive advantages to flagellate *Lactobacillus* species. Among the accepted motile species found in the *L. salivarius* clade, most have been isolated from environmental sources (Table S1), where the advantages of motility for nutrient acquisition and niche colonisation justify the associated metabolic costs of flagellum production and energization. *L. ruminis* however, is currently the only motile *Lactobacillus* species known to be autochthonous to the mammalian intestine, [29] and its potential production of flagella and therefore of flagellin, a well characterized MAMP *in vivo* is particularly relevant since it is known that flagellate commensal bacteria could have a significant effect on gut physiology [63], [64].

At least 43 genes are involved in the *Lactobacillus* flagellum-biogenesis and chemotaxis system, and these are chiefly found at a single locus in the *L. ruminis* genome. Although the deletion of genes involved in flagellum synthesis is known to be rapid in an environment where motility confers no evolutionary advantage [65], the *L. ruminis* ATCC25644 (non-motile, human isolate) and ATCC27782 (motile, bovine isolate) motility loci share 97% nucleotide identity over their entire length. The absence of gross deletion, despite a typically non-motile phenotype, suggests that *L. ruminis* strains isolated from humans may produce flagella and be restored to full motility under particular environmental condition(s), for example *in vivo*. The historical report of the original ATCC25644 strain being motile would support such a theory, although fully motile ATCC25644 cultures have not been achieved in the laboratory to date. Nevertheless, a motility phenotype of some ATCC25644 cells was partially acquired (tumbling, without runs) following passage through the murine GI tract. Thus, it could be possible that the composition of the MRS growth medium limits the motility of ATCC25644. However, when MRS motility agar plates were prepared with alternative protein, carbon and phosphate sources, full running motility was not imparted to this strain.

Because it was not possible to demonstrate full motility in ATCC25644, and also on the basis of genomic and transcriptomic data, we hereby propose a model to partially explain the genetic basis for the differential motility of *L. ruminis* strains (Figure S4). Although the genetic complement of the motility loci of the two *L. ruminis* genomes studied is very similar, the motility locus of *L. ruminis* ATCC27782 (motile) differs from the motility locus of *L. ruminis* ATCC25644 (non-motile) by harboring an extra flagellin gene. Significantly, it is this additional flagellin gene *fliC*2, and not *fliC*1 which is common to both genomes, that is expressed at high levels by the motile strain. We propose that the short palindromic sequence AGATCT (a known ATP-DnaA binding site in *E. coli* [60]) that was identified at the *L. ruminis fliC*1 promoter, might serve as a transcription-factor binding site which, when occupied, would prevent transcription from this promoter.

We also suggest that the presumptive regulator ANHS_518/LRC_15730 is under the control of σ^70′^ (LRC_04220/ANHS_51c), a sigma factor that is highly expressed in ATCC25644 (Table S3). *In silico* examination of the predicted secondary structure of ANHS_518/LRC_15730 identified potential for forming a partial leucine zipper DNA-binding domain at its carboxy-terminus. This protein has no characterized homologs and may represent an atypical class of flagellum regulator that binds to motility gene promoters and operators to repress their transcription. The extent of the proposed negative regulation by ANHS_518/LRC_15730 is unclear, and may include a master regulator, since all of the genes/transcription units in the motility locus of ATCC25644 are down-regulated.

The motility gene content and arrangement of the *L. ruminis* and *L. mali* motility loci are broadly similar. It seems likely that the conserved motility operon structure resulted from selection for efficient flagellum assembly, which is known to be hierarchically regulated at the level of transcription in many species [66]. Of note is the organization of the motility genes in a single, uninterrupted locus in *L. ruminis*, particularly since the motility genes of *B. subtilis,* [67] and a number of other motile *Firmicutes* are distributed around their respective genomes or their motility loci are interrupted by genes with no known role in motility (Forde et al., in preparation). The inclusion of genes for rhamnose utilization in the motility locus of *L. mali* may indicate that the flagellin proteins of this species are modified by glycosylation, since this sugar has been found as a post-translational modification of flagellin in other bacteria [68], [69].

Recently, draft *L. vini* [70] and *L. acidipiscis* [71], [72] genomes were published, and these also harbour motility genes. While the motility of *L. vini* has previously been acknowledged [73], *L. acidipiscis* has not been formally described as a motile species [74]. Thus, as more *Lactobacillus* genomes are sequenced, the true penetrance of motility among the lactobacilli will emerge. It will also be interesting to determine if motility genes are indeed confined to species of the *L. salivarius* phylogenetic clade which would have significant evolutionary implications.

The observed congruence of motility protein based phylogenetic trees and the 16S rRNA gene tree strongly suggests that the *L. ruminis* motility genes were inherited by vertical descent from the *L. ruminis-Carnobacterium-Enterococcus* last common ancestor. Deeper studies of gene and protein phylogeny would be required to better resolve the more ancestral origins of the *Lactobacillus* motility system.

In the mammalian gut, it has been shown that MAMPs of the commensal luminal bacteria are recognised by the host’s pattern recognition receptors (PRR), such as the TLRs [75]. Recognition of the commensal microbiota by host PRRs may contribute to homeostasis and protection from injury in the gut [75]. *Lactobacillus*-TLR engagement may influence expression of these receptors [36] and downstream signalling through them [21]. Although *Lactobacillus* engagement of TLRs 2, 4 and 9 has been well established in the literature [20], [21], the significance of *Lactobacillus* flagellin-TLR5 signalling had not been studied previously. Thus, the observation of a robust proinflammatory response of human IECs to flagellate *L. ruminis* cells and flagellin proteins is biologically significant, and the secretion of IL8 is consistent with flagellin-TLR5 interactions for other species [6]. Of note however, is a report describing how aflagellate *L. plantarum* BFE 1685 and *L. rhamnosus* GG could increase expression of TLR5 in HT-29 cells through an unknown mechanism [36]. Since *L. plantarum* and *L. rhamnosus* are both species that may be probiotic, the ability of *L. ruminis* to signal to TLR5 does not necessarily exclude this species from being used as a probiotic. For example, *L. ruminis* could contribute to basal stimulation of the innate immune system [75] and also sensitize IECs so that they respond better to flagellate pathogens.

In summary, we suggest that the flagellate, mammalian GI commensal *L. ruminis,* may serve as a model species for the study of motility among the lactobacilli, a trait that has been poorly characterized until now. This characterization of the innate immune response to flagellate *Lactobacillus* cells and *Lactobacillus* flagellin proteins has allowed us to predict the *in vivo* responses of the human host to this autochthonous GI bacterium. This immunological information may become particularly relevant if flagellate *Lactobacillus* species are to be developed for probiotic, pharmaceutical, or vaccination applications.

## Materials and Methods

### Bacterial Strains, Growth Conditions and Motility Evaluation

*Lactobacillus* strains (Table S2) were cultured anaerobically at 37°C (*L. ruminis*) or 30°C (*L. ghanensis, L. mali*, and *L. nagelii*) in MRS medium under anaerobic conditions for 48–96 hours. *E. coli* strains were grown aerobically (with agitation) at 37°C in LB broth supplemented with 100 µg/ml ampicillin and 34 µg/ml chloramphenicol.

Standard motility agar plates comprised MRS broth (Oxoid) supplemented with 0.3%–0.5% (w/v) agarose. MRS broth prepared from first principles followed the composition of standard MRS broth (Oxoid). Glucose was added separately after sterilization. The following substitutions were made to prepare MRS media (500 ml) with alternative protein, phosphate and carbon sources: 9 g of Bactocasitone or Bactopeptone in place of peptone (5 g) and Lab Lemco powder (4 g); 1 g of β-glycerophosphate in place of dipotassium hydrogen phosphate; 10 g of preferred carbohydrate in place of glucose. The motility plates were inoculated with a 10 µl aliquot of an overnight *L. ruminis* culture that had been concentrated by slow centrifugation and resuspension in 1 ml sterile PBS. The inoculum was allowed to dry onto the surface of the plate for 3 min before the plate was moved. Plates were incubated for at least 48 hours.

Culture motility was evaluated qualitatively by phase-contrast microscopy. Glass capillary tubes were filled with an aliquot of the bacterial culture to be tested. The capillary tube was placed on a heated microscope stage that was maintained at 37°C for evaluation of culture motility. When every bacterium in a field of view of the microscope was either running or tumbling and moving quickly, the culture was considered “very motile”. When most of the bacteria in a field of view were motile and moving at a moderate speed, the culture was considered “moderately motile”. A culture was considered “weakly motile” when most of the bacteria in a field of view were not motile but some motile cells were also present. If no motile bacteria were observed in the fields of view examined, the culture was considered “non-motile”. Motility could be restored to stationary phase *L. ruminis* cultures of bovine origin by the addition of fresh (1.5 vol) MRS broth followed by anaerobic incubation for 2–3 hours.

### Genome Sequencing, Assembly and Comparative Genomics

*L. ruminis* ATCC25644 and ATCC27782 genomes were each sequenced to at least 21-fold coverage by generation of 200,000 454-FLX reads of average length 125–150 nt, with 3 kb paired-end data and with a half lane of Illumina 3 kb mate-pair and 400 nt paired end libraries. The average Illumina read length was 38 nt. Genomes were assembled using GS Assembler (Roche) and MIRA. The assembly, automated annotation and manual curation of these genomes has been described elsewhere [59]. These *L. ruminis* genomes have been issued the following GenBank accession numbers: AFYE00000000.1, (ATCC25644) and CP003032.1 (ATCC27782).

The *L. mali* DSM20444^T^ genome was also subjected to *de novo* assembly using Velvet (version 0.7.58) [76]. Two-hundred contigs were assembled into 95 scaffolds with an N50 of 92,201 nt. A paired-end library consisting of 90 bp reads separated by 500 nt assisted the assembly. The draft *L. mali* genome was annotated using the Prokaryotic Genomes Automatic Annotation Pipeline (PGAAP) which is available on the NCBI website. An automated annotation of the motility contig (contig 1) was separately subjected to manual curation and improvement. Artemis Comparison Tool, [77] BLAST [78] were used to compare the *L. ruminis* and *L. mali* motility loci. This *L. mali* sequencing project has been assigned the BioProject number PRJNA84435 and GenBank accession number AKKT00000000.

### Flagellin Extraction

Flagella were recovered and flagellin was purified as outlined previously for *C. jejuni* [79] with modifications as described here. Motility was verified by light-microscopy. The entire culture volume (0.5–4 L) was centrifuged (1750×*g,* 20 mins, 4°C). Cell pellets were re-suspended in 80 ml of cold PBS. Cell-suspensions were homogenized for 30 seconds using a Waring commercial blender (Waring Blendor) at the high speed setting. Homogenization was repeated three times. Cell suspensions were incubated on ice for 30 seconds between blending. Cellular debris was removed by centrifugation, (10,000 *g,* 20 mins, 4°C). Crude flagellin protein was concentrated by ultracentrifugation, (100,000 *g*, 1 hour, 4°C). Resulting pellets were re-suspended in 100–200 µl of sterile-distilled water. Protein concentrations were determined using the Pierce BCA protein assay (ThermoScientific) according to manufacturer’s instructions. For amino terminal sequencing, flagellin proteins were transferred to Immobilon membrane. The first ten to fifteen amino-terminal residues were sequenced at Aberdeen Proteomics (Aberdeen, Scotland).

### Transmission Electron Microscopy

Electron microscopy was carried out at the EM facility, University College Cork, and at the University of Birmingham, UK. Specimens were negatively stained with ammonium molybdate solution, 0.25%–2%, pH 7.

To harvest and concentrate the bacteria without damaging the cells or the flagella, motile *L. ruminis* ATCC27782 cultures in early exponential phase were passed through a 0.2 µm filter (Millipore). Bacteria were recovered by rinsing the filter with 0.15 M NaCl. The bacterial suspension was centrifuged at 100×*g* for 45 min at room temperature, the supernatant was discarded and the cell pellet was gently re-suspended in 300 µl of 0.15 M NaCl. Non-motile *L. ruminis* ATCC25644 cells, also in early exponential phase, were harvested by centrifugation (660×*g*, 20 min; room temperature) and the cell pellet was resuspended in 300 µl of 0.15 M NaCl.

Copper grids were either floated on, or immersed in, the bacteria suspension for 30 s to 5 min. The grids were stained with an ammonium molybdate solution (0.25%−2%), pH 7 for 20 s. The 1% and 2% ammonium molybdate solutions were supplemented with a wetting agent (70 µg/ml bacitracin). Before and after staining, excess liquid was removed from the surface of the grid using filter paper. Grids were air-dried and were viewed using a transmission electron microscope (JEOL Transmission Electron Microscope (JEOL Ltd., Tokyo, Japan), (JEM 2000FXII at University College Cork), (JEOL JEM-1200EX at University of Birmingham).

### Epithelial Cell Response to *Lactobacillus* Flagella and Flagellin Proteins

Human intestinal epithelial cell lines, HT-29 (ATCC HTB-38); Caco-2 (ATCC HTB-37) and T84 (ATCC CCL-248) were cultured at 37°C in DMEM (Invitrogen) supplemented with 10% foetal calf serum and 1% penicillin and streptomycin (Stock concentrations of these antibiotics were 10,000 U/ml penicillin, 10 mg/ml streptomycin).

For routine stimulations, human IECs were seeded in triplicate at 2×10^5^ cells per well of a flat-bottomed 96 well tissue-culture plate and were incubated under standard conditions for 24 hours. Then, the IECs were exposed either to *L. ruminis* cells (MOI, 10∶1) or to *Lactobacillus* or *Salmonella* flagellin proteins (final concentration: 0.1 µg/ml) for twelve hours. The bacteria and protein stimuli were prepared in DMEM with 10% foetal calf serum and antibiotics. The bacteria were washed twice with PBS before being resuspended in the tissue culture medium.

The amount of IL8 secreted in response to these various stimuli were measured with either the ELISA human IL8 Duo Kit (R&D systems) or MSD (Meso Scale Discovery) immunoassay plates which were used according to the manufacturer’s instructions. Four parameter logistic curves were used to generate standard curves from which the IL8 concentrations of the supernatants harvested from stimulated IECs were derived.

For siRNA experiments, non-polarized HT-29 cells were seeded in triplicate at 2×10^5^ cells per well of a 96 well plate, in antibiotic free DMEM with 10% foetal calf serum. The plate was incubated under standard conditions overnight.

HT-29 cells were transfected with either siRNA targeting TLR5 or non-targeting control siRNA at a final concentration of 100 nM siRNA per well. The transfection was performed according to the manufacturer’s guidelines. Briefly, equal volumes of siRNA and OptiMEM transfection medium were mixed in the same tube. In a separate tube, DharmaFECT transfection reagent was diluted with OptiMEM medium. After 5 min incubation at room temperature, the contents of both tubes were mixed and incubated for 20 min at room temperature. Antibiotic free DMEM with added foetal bovine serum (FBS) was used to adjust the final volume of the transfection medium. A 100 µl aliquot of this transfection medium was added to the HT-29 cells in place of the antibiotic free medium that had bathed the cells overnight. The HT-29 cells were incubated in this transfection medium for 48 hours under standard conditions before stimulation with whole *L. ruminis* cells (2×10^6^ bacteria/well) resuspended in DMEM with 10% FBS and 1% penicillin/streptomycin.

Cell viability assays were performed using the “CellTiter-Glo® Luminescent Cell Viability Assay” (Promega) according to the manufacturer’s instructions.

The efficiency of siRNA gene silencing was investigated by qRT-PCR. Epithelial cell lysates were harvested after 12 hours of exposure to whole *L. ruminis* cells. PCR primers and probes were designed using the Universal ProbeLibray Assay Design Centre (https://www.roche-applied-science.com/sis/rtpcr/ulp/adc.jsp) (sequences in Table S5). β-actin (ACTB) was used as the calibrator gene. Amplification reactions were prepared with the FastStart TaqMan Probe Master kit (Roche), using 900 nM of each primer in a total volume of 10 µl. Reactions were performed in triplicate on the LightCycler 480 System (Roche) under the following cycling conditions: Pre-incubation: 1 cycle; 95°C, 10 min. Amplification: 45 cycles; 95°C, 10 s; 60°C, 45 s; 72°C, 1 s. Cooling: 1 cycle; 40°C, 30 s. Relative changes in gene expression were calculated according to the 2^−ΔΔCt^ method [80].

### Statistical Analyses

A one-tailed Mann-Whitney U test was used for statistical analysis of all cytokine data. Data scaling was applied where appropriate to normalize data from independent experiments. This scaling required the conversion of the IL8 concentration determined for each variable in a replicate experiment to a proportion. This was achieved by dividing the IL8 concentration calculated for each variable in a single experiment by the sum of IL8 secretion for all variables in the same experiment. Statistical tests were applied to these proportions.

### Expression and Purification of Recombinant *L. ruminis* ATCC25644 FliC1

An *L. ruminis* ATCC25644 *fliC*1 PCR product was engineered to include a SmaI and an XhoI restriction site at its 5′ and 3′ termini respectively. Primer sequences are given in (Table S5). The amplified product was restricted and ligated into the pGEX-6-P-3 expression vector (GE Healthcare), immediately downstream of the gene encoding GST. The vector was transformed into *E. coli* Top Ten for plasmid maintenance and into *E. coli* Rosetta 2(DE3) pLysS (Novagen) for expression.

The target GST-FliC1 fusion protein was isolated from inclusion bodies. Expression of the gene for the recombinant protein was induced by addition of 0.1 mM IPTG (final concentration), to a 50–75 ml *E. coli* Rosetta culture in the exponential growth phase (OD_600_ 0.4). The culture was incubated aerobically at 37°C with agitation for at least 16 hours. Cell pellets were harvested by centrifugation followed by an optional lysozyme 50 mg/ml treatment, and were eventually resuspended in 10 ml wash buffer (1×PBS; 1% Triton-X-100, 1 mM EDTA, pH 7.4).). The cell suspension was lysed by passing it through a French press (10,000 psi, twice). The inclusion bodies were recovered from the lysate by centrifugation (15,000 *g*, 30 min, 4°C). The pellet was resuspended in 10 ml wash buffer and the centrifugation step was repeated. The cell pellet was similarly twice washed in chilled water and was incubated overnight at 4°C in solubilization buffer (20 mM TrisHCl, 6 M Urea, 500 mM NaCl, 5 mM β-mercaptoethanol, pH 8). The remaining insoluble material was removed by centrifugation (18,000 *g*, 45 min, 4°C). The supernatant which contained the target protein was refolded by dialysis against a series of solutions containing 0.1 M Tris, 0.5 M arginine and decreasing concentrations (4 M –0 M) of urea. Dialysis against each solution took place for 24 hours at 4°C. Optionally, post-dialysis, the protein samples were washed with 3 volumes of 0.1 M Tris and concentrated with an Amicon 10 K ultra centrifugal filter (Millipore Ltd).

### Transcriptome Analysis

*L. ruminis* ATCC27782 and ATCC25644 were cultured anaerobically at 37°C for 15 hours in 20 ml aliquots of MRS media. Each culture was centrifuged at room temperature to harvest the cells that were immediately resuspended in 500 µl of RNAprotect Bacteria Reagent (Qiagen). Total RNA was isolated according to the protocol for difficult-to-lyse bacteria outlined in the RNAprotect Bacteria Reagent (Qiagen) handbook, but with an extended proteinase K incubation (40 mins). The RNeasy Mini kit (Qiagen) was used to complete the extraction procedure. Contaminating DNA was removed with the Turbo DNA-free kit (Ambion).

cDNA for microarray analysis was prepared by reverse transcribing 10 µg of total RNA using random nonomers (MWG-Biotech, Germany) and the ULS cDNA synthesis and labeling Kit (Kreatech). The details of probe hybridization, (60°C, 20 hours) microarray scanning and analysis are described elsewhere [59]. Genes with an expression ratio ≥5 and a p-value of ≤1.0×10^−4^, were considered significantly up or down regulated. The microarray data is available through the GEO website and has been assigned accession number, GSE31556.

For qRT-PCR, RNA was reverse transcribed using Superscript II (Invitrogen) kit and random primers. All reactions were performed in triplicate using the Lightcycler FastStart DNA Master^plus^ SYBR green I kit (Roche) and the LightCycler 480 System (Roche). Relative changes in gene expression were calculated according to the 2^−ΔΔCt^ method [80].

For several genes, qRT-PCR was performed to verify the result of the microarray analysis. cDNA was purified following overnight reverse transcription using the Illustra CyScribe GFX Purification Kit (GE Healthcare). The purified cDNA was quantified with a Nanodrop (Thermo Scientific). A 10 ng aliquot of cDNA was used as template per qRT-PCR reaction. Primers were used at a final concentration of 0.2 M per reaction. Primer sequences were designed for *groEL*, *fliM* and LRC_15730 (Table S5). The qRT-PCR program was as follows: Pre-incubation: 1 cycle; 95°C, 10 min. Amplification: 40 cycles; 95°C, 10 s; 59°C, 10 s; 72°C, 10 s. Melting curve: 1 cycle; 95°C, 5 s; 55°C, 1 min; 97°C, continuous. Cooling: 1 cycle; 40°C, 30 s.

To distinguish transcription from ATCC27782 flagellin genes *fliC*1 (LRC_15700) and *fliC*2 (LRC_15680), RNA from motile ATCC27782 cells was reverse transcribed as previously described. Primer pairs specific to each flagellin gene and *era* were designed (Table S5). The qRT-PCR program was as follows: Pre-incubation: 1 cycle; 95°C, 10 min. Amplification: 45 cycles; 95°C, 10 s; 65°C, 5 s;72°C, 5 s. Melting curve: 1 cycle; 95°C, 5 s; 55°C, 1 min; 97°C, continuous. Cooling: 1 cycle; 40°C, 30 s.

### 5′ Rapid Amplification of cDNA Ends

The transcription start sites of three target genes were determined using the 5′ RACE 2^nd^ generation kit (Roche) according to the manufacturer’s protocol. RNA was extracted from exponential phase *L. ruminis* cultures (OD_600_ ∼0.8) as previously described. RNA from ATCC25644 was reverse transcribed using primers specific for the potential negative regulator (ANHS_518) and the sigma^70′^ (ANHS_51c). RNA from ATCC27782 was reverse transcribed using flagellin specific primers (Table S5). The cDNA generated was purified and tailed using the High Pure Purification kit (Roche). A series of subsequent PCR reactions, in which the product of an earlier reaction provided template for the next reaction, generated products that when sequenced, facilitated identification of the desired transcription start sites. DreamTaq (Fermentas) was used for PCR reactions, and the T_A_ was 50°C.

### Phylogenetic Analyses and Protein Alignments

Motility proteins from UniProt and NCBI protein database (accession numbers given in Table S6) were identified in each species using a combination of existing annotation, BLAST searches [78] and gene order. Protein sequences were aligned using MUSCLE [81]. Any multiple sequence alignment columns that contained at least one gap were removed. Thus the sequences included in the resulting file were all exactly the same length. An appropriate substitution model for tree construction was selected according to the output of Modelgenerator [82]. Trees were constructed using PHYML [83] with 100 bootstrap replications. 16S rRNA gene trees were generated with TreeBuilder on the Ribosomal database project website [84]. A clade was considered to be strongly supported if its bootstrap value was ≥90.

### Bioinformatic Analysis of ANHS518/LRC_15730

The ANHS_518 and LRC_15730 protein sequences were subjected to BLASTp, PSI-BLASTp analyses on the NCBI website. These sequences were also used as Interproscan and Pfam queries to identify if any conserved domains were present.

### Generation of Rifampicin Tagged *L. ruminis* Strains, Mouse Trial and Motility Evalutation

Rifampicin-resistant *L. ruminis* ATCC25644 and *L. ruminis* ATCC27782 variants were produced by serially subculturing these strains in MRS media with increasing concentrations of rifampicin until bacteria resistant to 200 µg/ml rifampicin were recovered. During the mouse trial, these bacteria were cultured in MRS broth (20 ml) with 200 µg/ml rifampicin daily. The bacterial cultures centrifuged to harvest the bacteria, which were washed and resuspended in 2 ml sterile PBS. In addition to a standard rodent diet, two groups of five BALB/c mice (6–8 weeks old) were fed a 200 µl aliquot of either rifampicin resistant ATCC25644 or ATCC27782 by oral gavage once daily for five days. Fecal pellets from each group were collected daily. Faecal pellets from each group were pooled, homogenized and serially diluted in PBS. The rifampicin resistant strains were recovered by plating the various dilutions on MRS agar plates containing 50 µg/ml rifampicin. Plates were incubated anaerobically at 37°C for two days. Single colonies recovered from these plates were added to 2 ml MRS broth aliquots containing 50 µg/ml rifampicin and were incubated anaerobically at 37°C for 24 hours, until the cultures became turbid. Glycerol stocks were prepared for each culture. For motility screening, these stocks were used to inoculate MRS broth containing 50 µg/ml rifampicin. The motility of the *L. ruminis* ATCC25644 rifampicin resistant strains that were recovered was evaluated by phase-contrast microscopy and flagellin isolation followed by Western blotting.

For Western blotting, proteins were transferred from an SDS-PAGE gel to PVDF membrane using an EC140 mini-blot apparatus according to the manufacturer’s instructions. Briefly, a gel sandwich was prepared using sponges and filter paper soaked in Towbin buffer (25 mM Tris, 192 mM glycine, pH 8.3). The PVDF membrane was activated in methanol before use. Transfer took place at 15 V constant voltage for 1 hour. The membrane was blocked with a solution of 1% skimmed milk in 1×TBS (20 mM Tris, 0.15 M NaCl, pH 7.6). An anti-*L. ruminis* flagellin primary antibody was used at a concentration of 1 µg/ml. This custom designed antibody was raised in rabbits by GenScript, USA. The antibody was designed to target the *L. ruminis* flagellin sequence GLTQAKRNAQDGISC. The secondary antibody used was an anti-rabbit IgG peroxidise conjugate. The presence of flagellin was confirmed by colorimetric development of each blot, which was achieved by the addition of 30 mg chloronapthol dissolved in 10 ml methanol to 50 ml 1×TBS with 33 µl hydrogen peroxide. A blue/black result confirmed the presence of flagellin protein.

The identity of the tumbling ATCC25644 strains recovered was confirmed by strain-specific PCR, 16S rRNA sequencing and API carbohydrate utilization profiling.

*L. ruminis* ATCC25644 specific primer sequences (1054561-1055463; 653884-654800; 626252-627921; 380111-380820; 1654861-1656335; 1338430-1339559; 644444-645201, Table S5) were designed to target ATCC25644 genome sequences that were not present in the ATCC27782 genome. PCR was performed using BioTaq (Bioline), using a 50°C annealing temperature, and a one minute extension time.

The ATCC25644 16S rRNA gene product for sequencing was generated by PCR using standard primers (27F and 1492R, Table S5). PCR was performed using a 50°C annealing temperature.

A set of API50CH “Research strips for investigation carbohydrate metabolism in bacteria” (Biomerieux), was inoculated with the tumbling ATCC25644 according to the manufacturers instructions. Carbohydrate utilization was evaluated after 72 hours anaerobic incubation at 37°C.

## Supporting Information

Figure S1 **Genetic organization of the** ***L. ruminis*** **ATCC27782 motility locus.** Motility genes are arranged contiguously and span 48 Kb of the genome. MCP  =  Methyl accepting chemotaxis protein. Locus tags are given below each gene arrow. The glycosyltransferase (LRC_15690) colored gray is frameshifted. The additional *fliC*2 gene (LRC_15680) present in ATCC27782, but absent from the ATCC25644 motility locus is colored purple. The LRC_15730 gene, (shown in red here), is a homolog of ANHS_518, the only gene at the *L. ruminis* ATCC25644 motility locus to be differentially transcribed in the non-motile strain.
(TIF)

Figure S2 **Artemis Comparison Tool (ACT) alignment of** ***L. ruminis*** **ATCC27782 and** ***L. mali*** **DSM20444^T^ motility loci.** The *L. ruminis* motility locus (top) is aligned to the *L. mali* motility locus (bottom) using tBLASTx. Red lines indicate regions of similar sequence with the same orientation in both genomes. Blue lines indicate regions of similarity sequence that have an inverted configuration. A large, 11.8 kb insertion in the *L. mali* locus relative to the *L. ruminis* motility locus is also evident.
(TIF)

Figure S3 **16S rRNA gene tree and motility protein based phylogenetic trees.** Trees were constructed using PHYML. Bootstrap values are given at each node.
(PDF)

Figure S4 **Proposed model for regulation of flagellum biogenesis.** Sigma 70′ is responsible for transcription of ANHS_518/LRC_15730, which acts via an unknown mechanism to inhibit transcription of the genes regulating flagellum biogenesis. Genes shown in red and green are upregulated in ATCC25644 and ATCC27782 respectively during exponential phase, when ATCC27782 is motile. Genes shown in blue are not differentially transcribed. The nucleotide sequence immediately upstream of *L. ruminis* flagellin genes and the putative negative regulator, ANHS_518 are shown. Start codons are coloured green. Predicted −10 and −35 sequences are blue. The +1 transcription start sites mapped by 5′ RACE are orange. Likely ribosome binding sites are underlined.
(TIF)

Figure S5 **Characterization of the immune responses induced by native and recombinant** ***Lactobacillus*** **flagellin proteins in epithelial cell lines.** A: HT-29 cells secrete IL8 in response to the native flagellin proteins of various *Lactobacillus* species as indicated. Flagellin was added at a final concentration of 0.1 µg/ml. Boxplots show the median values and interquartile range based on data from six experimental replicates. A one-tailed Mann-Whitney U test was applied to calculate statistical significance. The data upon which this graph is based were converted to proportions as described in Materials and Methods. B: T84 cells secrete IL8 in response to the recombinant GST-ATCC25644 flagellin protein. Graphs show median values and interquartile ranges. For “untreated” and “GST”, n = 5. For the recombinant protein, n = 7. A one-tailed Mann-Whitney U test was applied to calculate statistical significance.
(TIF)

Figure S6 **Failure of altered growth media to impart motility to** ***L. ruminis*** **ATCC25644 on semi-solid agar plates.** A, B: Standard (A) or half-strength (B) MRS (Oxoid) inoculated with ATCC25644. C, D: MRS prepared from first principles with alternative phosphate sources, β-glycerophosphate (C) or K2HPO4 (D) and inoculated with ATCC25644. E, F: MRS prepared from first principles with alternative protein sources, Bactocasitone (E) and Bactopeptone (F) and inoculated with ATCC25644. G, H: MRS prepared from first principles with alternative carbohydrate sources, P95 (G) and Synergy I (H) and inoculated with ATCC25644. I, J: MRS prepared from first principles with uracil (0.005 g/500 ml) inoculated with ATCC25644 (I) or ATCC27782 (J). These photographs are of motility plates that were inoculated on different days. *L. ruminis* ATCC25644 is not motile under any of these conditions. *L. ruminis* ATCC27782 (J) is representative of a motile culture on semi-solid agar, and can be seen to cover the entire plate surface.
(TIF)

Table S1 **Origin and phylogeny of motile** ***Lactobacillus*** **species described to date.**
(DOC)

Table S2 ***Lactobacillus*** **strains and species used in this study.**
(DOC)

Table S3 **Closest homologs of** ***L. ruminis*** **motility proteins.**
(DOC)

Table S4 **Expression analysis of flagellum biogenesis and chemotaxis genes in** ***L. ruminis*** **derived by type I microarray.** Values tabulated are expression ratios of ATCC27782 relative to ATCC25644. Values in parentheses represent relative fold differences in expression of ATCC25644 relative to ATCC27782. Relative data for qRT-PCR are also shown. Genes with at least 5 fold relative difference in expression and P-values <1.0×10^−4^ are in bold-face. ^†^ Normalized to expression level of *fliC*2, ATCC27782. ^‡^ Microarray probes for *fliC* could not distinguish between *fliC1* and *fliC2*, although qRT-PCR shows only *fliC*2 is expressed. ND = No data.
(DOC)

Table S5 **Primers used in this study.**
(DOC)

Table S6 **Uniprot and NCBI accession numbers for motility proteins used for phylogenetic analysis.**
(DOC)

Movie S1 **Microscopic observation of motile** ***L. ruminis*** **ATCC27782 cells.** Bacteria were grown in MRS broth and were visualised using a light-microscope. Video was recorded with a USB eyepiece camera. Motile single cells and chains of motile cells are visible.
(WMV)

Movie S2 **Microscopic observation of tumbling** ***L. ruminis*** **ATCC25644 cells in MRS broth.** Video was recorded with a USB eyepiece camera attached to a phase-contrast microscope.
(WMV)

## References

[1] Yuan J, Fahrner KA, Turner L, Berg HC (2010). Asymmetry in the clockwise and counterclockwise rotation of the bacterial flagellar motor.. Proc Natl Acad Sci U S A.

[2] Macnab RM (2003). How bacteria assemble flagella.. Annu Rev Microbiol.

[3] Hayashi F, Smith KD, Ozinsky A, Hawn TR, Yi EC (2001). The innate immune response to bacterial flagellin is mediated by Toll-like receptor 5.. Nature.

[4] Letran S, Lee S, Atif S, Flores-Langarica A, Uematsu S (2011). TLR5-deficient mice lack basal inflammatory and metabolic defects but exhibit impaired CD4 T cell responses to a flagellated pathogen.. J Immunol.

[5] Gewirtz AT, Navas TA, Lyons S, Godowski PJ, Madara JL (2001). Cutting edge: bacterial flagellin activates basolaterally expressed TLR5 to induce epithelial proinflammatory gene expression.. J Immunol.

[6] Tallant T, Deb A, Kar N, Lupica J, de Veer M (2004). Flagellin acting via TLR5 is the major activator of key signaling pathways leading to NF-kappaB and proinflammatory gene program activation in intestinal epithelial cells.. BMC Microbiol.

[7] Walter J, Tannock GW (2005). The microecology of lactobacilli in the gastrointestinal tract..

[8] Lane MC, Alteri CJ, Smith SN, Mobley HLT (2007). Expression of flagella is coincident with uropathogenic *Escherichia coli* ascension to the upper urinary tract.. Proc Natl Acad Sci U S A.

[9] Lemon KP, Higgins DE, Kolter R (2007). Flagellar motility is critical for *Listeria monocytogenes* biofilm formation.. J Bacteriol.

[10] Houry A, Briandet R, Aymerich S, Gohar M (2010). Involvement of motility and flagella in *Bacillus cereus* biofilm formation.. Microbiology.

[11] Konkel ME, Klena JD, Rivera-Amill V, Monteville MR, Biswas D (2004). Secretion of virulence proteins from *Campylobacter jejuni* is dependent on a functional flagellar export apparatus.. J Bacteriol.

[12] Macnab RM, Neidhardt FC, Curtiss III R, Ingraham JL, Lin ECC, Brooks Low K (1996). Flagella and motility..

[13] Grundling A, Burrack L, Bouwer H, Higgins DE (2004). *Listeria monocytogenes* regulates flagellar motility gene expression through MogR, a transcriptional repressor required for virulence.. Proc Natl Acad Sci U S A.

[14] Peel M, Donachie W, Shaw A (1988). Temperature-dependent expression of flagella of *Listeria monocytogenes* studied by electron microscopy, SDS-PAGE and western blotting.. J Gen Microbiol.

[15] Zhou K, Kanai R, Lee P, Wang H, Modis Y (2012). Toll-like receptor 5 forms asymmetric dimers in the absence of flagellin.. J Struct Biol.

[16] Yoon SI, Kurnasov O, Natarajan V, Hong M, Gudkov AV (2012). Structural basis of TLR5-flagellin recognition and signaling.. Science.

[17] Smith KD, Andersen-Nissen E, Hayashi F, Strobe K, Bergman MA (2003). Toll-like receptor 5 recognizes a conserved site on flagellin required for protofilament formation and bacterial motility.. Nat Immunol.

[18] Andersen-Nissen E, Smith KD, Bonneau R, Strong RK, Aderem A (2007). A conserved surface on Toll-like receptor 5 recognizes bacterial flagellin.. J Exp Med.

[19] Andersen-Nissen E, Smith KD, Strobe KL, Barrett SL, Cookson BT (2005). Evasion of Toll-like receptor 5 by flagellated bacteria.. Proc Natl Acad Sci U S A.

[20] Lebeer S, Vanderleyden J, De Keersmaecker SC (2008). Genes and molecules of lactobacilli supporting probiotic action.. Microbiol Mol Biol Rev.

[21] Wells J (2011). Immunomodulatory mechanisms of lactobacilli.. Microb Cell Fact 10.

[22] Felis GE, Dellaglio F (2007). Taxonomy of lactobacilli and bifidobacteria.. Curr Issues Intest Microbiol.

[23] Torriani S, Van Reenen GA, Klein G, Reuter G, Dellaglio F (1996). *Lactobacillus curvatus* subsp. *curvatus* subsp. nov. and *Lactobacillus curvatus* subsp. *melibiosus* subsp. nov. and *Lactobacillus sake* subsp. *sake* subsp. nov. and *Lactobacillus sake* subsp. *carnosus* subsp. nov., new subspecies of *Lactobacillus curvatus* Abo-Elnaga and Kandler 1965 and *Lactobacillus sake* Katagiri, Kitahara, and Fukami 1934 (Klein, et al. 1996, emended descriptions), respectively.. Int J Syst Bacteriol.

[24] Kimura K, McCartney AL, McConnell MA, Tannock GW (1997). Analysis of fecal populations of bifidobacteria and lactobacilli and investigation of the immunological responses of their human hosts to the predominant strains.. Appl Environ Microbiol.

[25] Kimura K, Nishio T, Mizoguchi C, Koizumi A (2010). Analysis of the composition of lactobacilli in humans.. Bioscience Microflora.

[26] Dal Bello F, Hertel C (2006). Oral cavity as natural reservoir for intestinal lactobacilli.. Syst Appl Microbiol.

[27] Rinttila T, Kassinen A, Malinen E, Krogius L, Palva A (2004). Development of an extensive set of 16S rDNA-targeted primers for quantification of pathogenic and indigenous bacteria in faecal samples by real-time PCR.. J Appl Microbiol.

[28] Stsepetova J, Sepp E, Kolk H, Loivukene K, Songisepp E (2011). Diversity and metabolic impact of intestinal *Lactobacillus* species in healthy adults and the elderly.. Br J Nutr.

[29] Tannock GW, Munro K, Harmsen HJ, Welling GW, Smart J (2000). Analysis of the fecal microflora of human subjects consuming a probiotic product containing *Lactobacillus rhamnosus* DR20.. Appl Environ Microbiol.

[30] Maukonen J, Matto J, Suihko ML, Saarela M (2008). Intra-individual diversity and similarity of salivary and faecal microbiota.. J Med Microbiol.

[31] Dommels YE, Kemperman RA, Zebregs YE, Draaisma RB, Jol A (2009). Survival of *Lactobacillus reuteri* DSM 17938 and *Lactobacillus rhamnosus* GG in the human gastrointestinal tract with daily consumption of a low-fat probiotic spread.. Appl Environ Microbiol.

[32] Kankainen M, Paulin L, Tynkkynen S, von Ossowski I, Reunanen J (2009). Comparative genomic analysis of *Lactobacillus rhamnosus* GG reveals pili containing a human- mucus binding protein.. Proc Natl Acad Sci U S A.

[33] Jakava-Viljanen M, Avall-Jaaskelainen S, Messner P, Sleytr UB, Palva A (2002). Isolation of three new surface layer protein genes (*slp*) from *Lactobacillus brevis* ATCC14869 and characterization of the change in their expression under aerated and anaerobic conditions.. J Bacteriol.

[34] Sun Z, Kong J, Hu S, Kong W, Lu W (2012). Characterization of a S-layer protein from *Lactobacillus crispatus* K313 and the domains responsible for binding to cell wall and adherence to collagen.. Appl Microbiol Biotechnol Epub ahead of print.

[35] Snyder LAS, Loman NJ, Futterer K, Pallen MJ (2009). Bacterial flagellar diversity and evolution: seek simplicity and distrust it?. Trends Microbiol.

[36] Vizoso Pinto MG, Rodriguez Gomez M, Seifert S, Watzl B, Holzapfel WH (2009). Lactobacilli stimulate the innate immune response and modulate the TLR expression of HT29 intestinal epithelial cells *in vitro*.. Int J Food Microbiol.

[37] Kajikawa A, Nordone SK, Zhang L, Stoeker LL, LaVoy AS (2011). Dissimilar properties of two recombinant *Lactobacillus acidophilus* strains displaying *Salmonella* FliC with different anchoring motifs.. Appl Environ Microbiol.

[38] Kajikawa A, Igimi S (2010). Innate and acquired immune responses induced by recombinant *Lactobacillus casei* displaying flagellin-fusion antigen on the cell-surface.. Vaccine.

[39] Heilig HG, Zoetendal EG, Vaughan EE, Marteau P, Akkermans AD (2002). Molecular diversity of *Lactobacillus* spp. and other lactic acid bacteria in the human intestine as determined by specific amplification of 16S ribosomal DNA.. Appl Environ Microbiol.

[40] Delgado S, Suarez A, Otero L, Mayo B (2004). Variation of microbiological and biochemical parameters in the faeces of two healthy people over a 15 day period.. Eur J Nutr.

[41] Reuter G (2001). The *Lactobacillus* and *Bifidobacterium* microflora of the human intestine: composition and succession.. Curr Issues Intest Microbiol.

[42] Sharpe ME, Latham MJ, Garvie EI, Zirngibl J, Kandler O (1973). Two new species of *Lactobacillus* isolated from the bovine rumen, *Lactobacillus ruminis* sp. nov. and *Lactobacillus vitulinus* sp. nov.. J Gen Microbiol.

[43] Krause DO, Smith WJ, Conlan LL, Gough JM, Williamson MA (2003). Diet influences the ecology of lactic acid bacteria and *Escherichia coli* along the digestive tract of cattle: neural networks and 16S rDNA.. Microbiology.

[44] Al Jassim RA (2003). *Lactobacillus ruminis* is a predominant lactic acid producing bacterium in the caecum and rectum of the pig.. Lett Appl Microbiol.

[45] Yin Q, Zheng Q (2005). Isolation and identification of the dominant *Lactobacillus* in gut and faeces of pigs using carbohydrate fermentation and 16S rDNA analysis.. J Biosci Bioeng.

[46] Kandler O, Weiss N, Sneath PHA, Mair NS, Sharpe ME, Holt JG (1986). Regular, nonsporing Gram positive rods, Bergey’s manual of sytematic bacteriology..

[47] Lerche M, Reuter G (1960). A contribution to the method of isolation and differentiation of aerobic “lactobacilli” (Genus “*Lactobacillus Beijerinck*”).. Zentralbl Bakteriol.

[48] Carr JG, Davies PA (1970). Homofermentative lactobacilli of ciders including *Lactobacillus mali* nov. spec.. J Appl Bacteriol.

[49] Kaneuchi C, Seki M, Komagata K (1988). Taxonomic study of *Lactobacillus mali* Carr and Davis 1970 and related Strains: Validation of *Lactobacillus mali* Carr and Davis 1970 over *Lactobacillus yamanashiensis* Nonomura 1983.. Int J Syst Evol Microbiol.

[50] Carr JG, Davies PA, Dellaglio F, Vescovo M, Williams AW (1977). The relationship between *Lactobacillus mali* from cider and *Lactobacillus yamanashiensis* from wine.. J Appl Microbiol.

[51] Dicks LMT, Endo A (2009). Taxonomic status of lactic acid bacteria in wine and key characteristics to differentiate species.. S Afr J Enol Vitic.

[52] Couto JA, Campos FM, Figueiredo AR, Hogg TA (2006). Ability of lactic acid bacteria to produce volatile phenols.. Am J Enol Vitic.

[53] Buron N, Coton M, Desmarais C, Ledauphin J, Guichard H (2011). Screening of representative cider yeasts and bacteria for volatile phenol-production ability.. Food Microbiol.

[54] Coton M, Romano A, Spano G, Ziegler K, Vetrana C (2010). Occurrence of biogenic amine-forming lactic acid bacteria in wine and cider.. Food Microbiol.

[55] Landete JM, Ferrer S, Pardo I (2007). Biogenic amine production by lactic acid bacteria, acetic bacteria and yeast isolated from wine.. Food Control.

[56] Seto A, Saito Y, Matsushige M, Kobayashi H, Sasaki Y (2006). Effective cellulose production by a coculture of *Gluconacetobacter xylinus* and *Lactobacillus mali*.. Appl Microbiol Biotechnol.

[57] Morishita T, Tamura N, Makino T, Kudo S (1999). Production of menaquinones by lactic acid bacteria.. J Dairy Sci.

[58] Chain PS, Grafham DV, Fulton RS, Fitzgerald MG, Hostetler J (2009). Genomics. Genome project standards in a new era of sequencing.. Science.

[59] Forde BM, Neville BA, O’Donnell MM, Riboulet-Bisson E, Claesson MJ (2011). Genome sequences and comparative genomics of two *Lactobacillus ruminis* strains from the bovine and human intestinal tracts.. Microb Cell Fact 10 S13.

[60] Speck C, Weigel C, Messer W (1999). ATP- and ADP-dnaA protein, a molecular switch in gene regulation.. EMBO J.

[61] McCracken A, Turner M, Giffard P, Hafner L, Timms P (2000). Analysis of promoter sequences from *Lactobacillus* and *Lactococcus* and their activity in several *Lactobacillus* species.. Arch Microbiol.

[62] Saad N, Urdaci M, Vignoles C, Chaignepain S, Tallon R (2009). *Lactobacillus plantarum* 299v surface-bound GAPDH: a new insight into enzyme cell walls location.. J Microbiol Biotechnol.

[63] Vijay-Kumar M, Aitken JD, Carvalho FA, Cullender TC, Mwangi S (2010). Metabolic syndrome and altered gut microbiota in mice lacking Toll-like receptor 5.. Science.

[64] Sitaraman SV, Klapproth JM, Moore DA, 3rd, Landers C, Targan S, et al (2005). Elevated flagellin-specific immunoglobulins in Crohn’s disease.. Am J Physiol Gastrointest Liver Physiol.

[65] Langridge G, Phan M-D, Turner D, Perkins T, Parts L (2009). Simultaneous assay of every *Salmonella* Typhi gene using one million transposon mutants.. Genome Res.

[66] Smith TG, Hoover TR (2009). Deciphering bacterial flagellar gene regulatory networks in the genomic era.. Adv Appl Microbiol.

[67] Kunst F, Ogasawara N, Moszer I, Albertini AM, Alloni G (1997). The complete genome sequence of the gram-positive bacterium *Bacillus subtilis*.. Nature.

[68] Schirm M, Arora SK, Verma A, Vinogradov E, Thibault P (2004). Structural and genetic characterization of glycosylation of type a flagellin in *Pseudomonas aeruginosa*.. J Bacteriol.

[69] Takeuchi K, Ono H, Yoshida M, Ishii T, Katoh E (2007). Flagellin glycans from two pathovars of *Pseudomonas syringae* contain rhamnose in D and L configurations in different ratios and modifided 4-amino-4,6-dideoxyglucose.. J Bacteriol.

[70] Luckwu de Lucena BT, Silva GG, Manoel Dos Santos B, Dias GM, Amaral GR (2012). Genome sequences of the ethanol-tolerant *Lactobacillus vini* strains LMG 23202T and JP7.8.9.. J Bacteriol.

[71] Kim DS, Choi SH, Kim DW, Kim RN, Nam SH (2011). Genome sequence of *Lactobacillus cypricasei* KCTC 13900.. J Bacteriol.

[72] Naser SM, Vancanneyt M, Hoste B, Snauwaert C, Swings J (2006). *Lactobacillus cypricasei* Lawson, et al. 2001 is a later heterotypic synonym of *Lactobacillus acidipiscis* Tanasupawat, et al. 2000.. Int J Syst Evol Microbiol.

[73] Rodas AM, Chenoll E, Macian MC, Ferrer S, Pardo I (2006). *Lactobacillus vini* sp. nov., a wine lactic acid bacterium homofermentative for pentoses.. Int J Syst Evol Microbiol.

[74] Tanasupawat S, Shida O, Okada S, Komagata K (2000). *Lactobacillus acidipiscis* sp. nov. and *Weissella thailandensis* sp. nov., isolated from fermented fish in Thailand.. Int J Syst Evol Microbiol 50 Pt.

[75] Rakoff-Nahoum S, Paglino J, Eslami-Varzaneh F, Edberg S, Medzhitov R (2004). Recognition of commensal microflora by toll-like receptors is required for intestinal homeostasis.. Cell.

[76] Zerbino DR, Birney E (2008). Velvet: algorithms for de novo short read assembly using de Bruijn graphs.. Genome Res.

[77] Carver TJ, Rutherford KM, Berriman M, Rajandream MA, Barrell BG (2005). ACT: the Artemis Comparison Tool.. Bioinformatics.

[78] Altschul SF, Gish W, Miller W, Myers EW, Lipman DJ (1990). Basic local alignment search tool.. J Mol Biol.

[79] Logan SM, Trust TJ (1983). Molecular identification of surface protein antigens of *Campylobacter jejuni*.. Infect Immun.

[80] Livak KJ, Schmittgen TD (2001). Analysis of relative gene expression data using real-time quantitative PCR and the 2(-Delta Delta C(T)) Method.. Methods.

[81] Edgar RC (2004). MUSCLE: multiple sequence alignment with high accuracy and high throughput.. Nucleic Acids Res.

[82] Keane TM, Creevey CJ, Pentony MM, Naughton TJ, McLnerney JO (2006). Assessment of methods for amino acid matrix selection and their use on empirical data shows that ad hoc assumptions for choice of matrix are not justified.. BMC Evol Biol.

[83] Guindon S, Gascuel O (2003). A simple, fast, and accurate algorithm to estimate large phylogenies by maximum likelihood.. Syst Biol.

[84] Cole JR, Chai B, Farris RJ, Wang Q, Kulam-Syed-Mohideen AS (2007). The ribosomal database project (RDP-II): introducing myRDP space and quality controlled public data.. Nucleic Acids Res.

