# Supplementary figures and images for: Characterization of Pro-Inflammatory Flagellin Proteins Produced by Lactobacillus ruminis and Related Motile Lactobacilli
Source: PLoS One. 2012 Jul 10;7(7):e40592. doi: 10.1371/journal.pone.0040592 (PMC3393694; doi:10.1371/journal.pone.0040592)

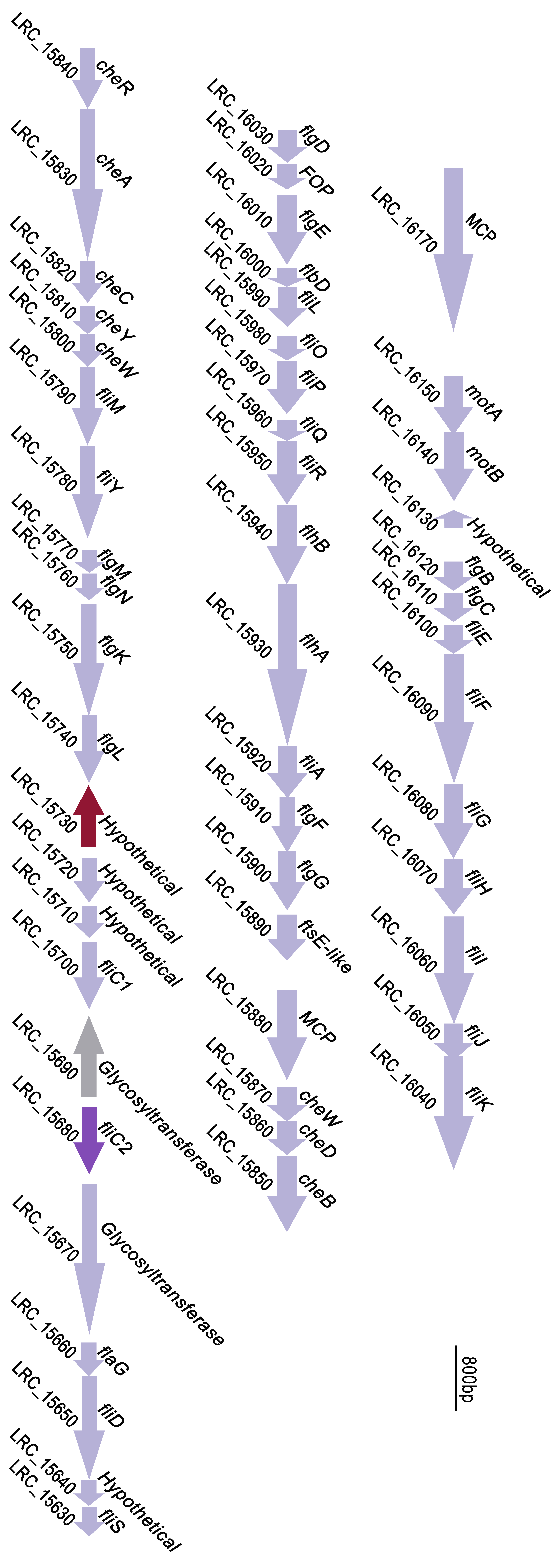

Supplement: Figure S1 — Genetic organization of the L. ruminis ATCC27782 motility locus. Motility genes are arranged contiguously and span 48 Kb of the genome. MCP = Methyl accepting chemotaxis protein. Locus tags are given below each gene arrow. The glycosyltransferase (LRC_15690) colored gray is frameshifted. The additional fliC2 gene (LRC_15680) present in ATCC27782, but absent from the ATCC25644 motility locus is colored purple. The LRC_15730 gene, (shown in red here), is a homolog of ANHS_518, the only gene at the L. ruminis ATCC25644 motility locus to be differentially transcribed in the non-motile strain. (TIF) [file pone.0040592.s001.tif]

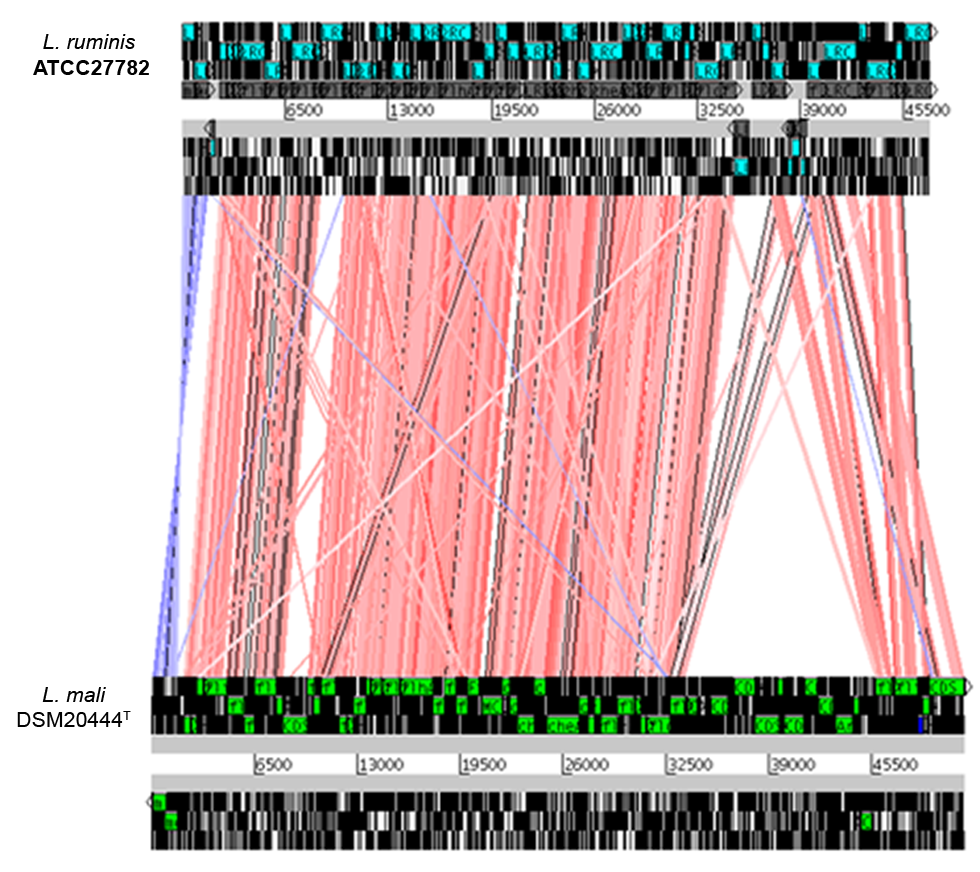

Supplement: Figure S2 — Artemis Comparison Tool (ACT) alignment of L. ruminis ATCC27782 and L. mali DSM20444T motility loci. The L. ruminis motility locus (top) is aligned to the L. mali motility locus (bottom) using tBLASTx. Red lines indicate regions of similar sequence with the same orientation in both genomes. Blue lines indicate regions of similarity sequence that have an inverted configuration. A large, 11.8 kb insertion in the L. mali locus relative to the L. ruminis motility locus is also evident. (TIF) [file pone.0040592.s002.tif]

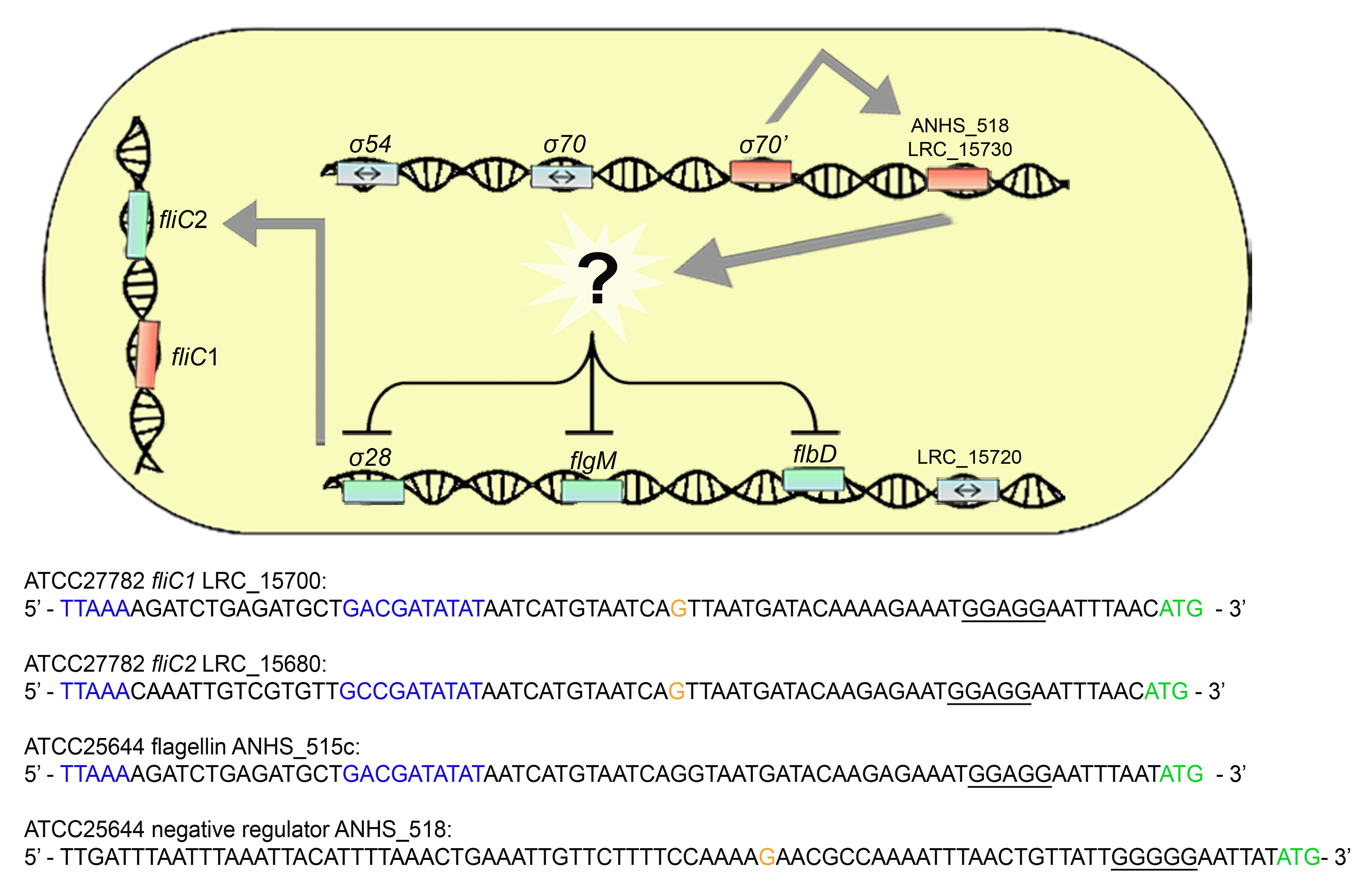

Supplement: Figure S4 — Proposed model for regulation of flagellum biogenesis. Sigma 70′ is responsible for transcription of ANHS_518/LRC_15730, which acts via an unknown mechanism to inhibit transcription of the genes regulating flagellum biogenesis. Genes shown in red and green are upregulated in ATCC25644 and ATCC27782 respectively during exponential phase, when ATCC27782 is motile. Genes shown in blue are not differentially transcribed. The nucleotide sequence immediately upstream of L. ruminis flagellin genes and the putative negative regulator, ANHS_518 are shown. Start codons are coloured green. Predicted −10 and −35 sequences are blue. The +1 transcription start sites mapped by 5′ RACE are orange. Likely ribosome binding sites are underlined. (TIF) [file pone.0040592.s004.tif]

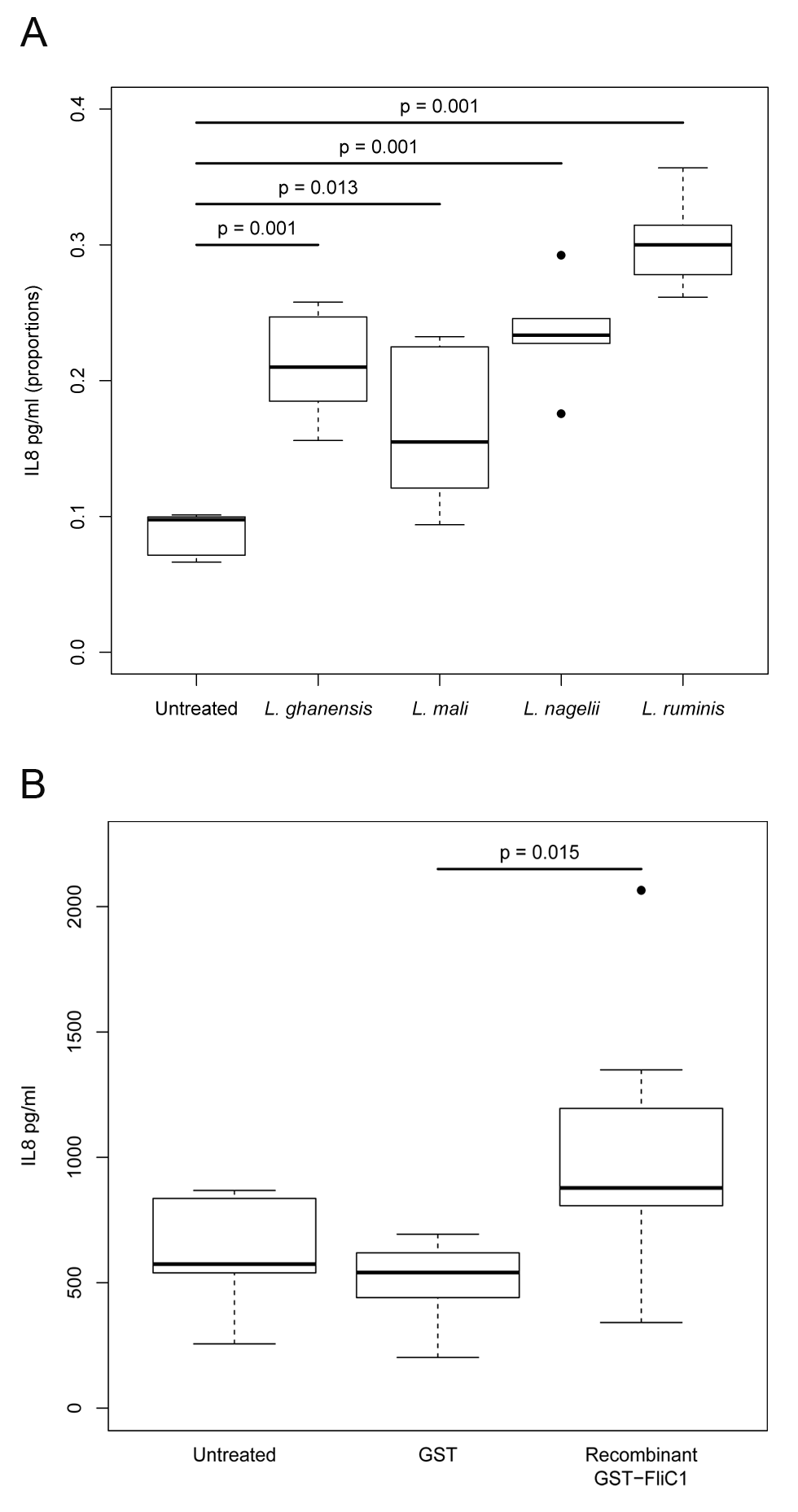

Supplement: Figure S5 — Characterization of the immune responses induced by native and recombinant Lactobacillus flagellin proteins in epithelial cell lines. A: HT-29 cells secrete IL8 in response to the native flagellin proteins of various Lactobacillus species as indicated. Flagellin was added at a final concentration of 0.1 µg/ml. Boxplots show the median values and interquartile range based on data from six experimental replicates. A one-tailed Mann-Whitney U test was applied to calculate statistical significance. The data upon which this graph is based were converted to proportions as described in Materials and Methods. B: T84 cells secrete IL8 in response to the recombinant GST-ATCC25644 flagellin protein. Graphs show median values and interquartile ranges. For “untreated” and “GST”, n = 5. For the recombinant protein, n = 7. A one-tailed Mann-Whitney U test was applied to calculate statistical significance. (TIF) [file pone.0040592.s005.tif]

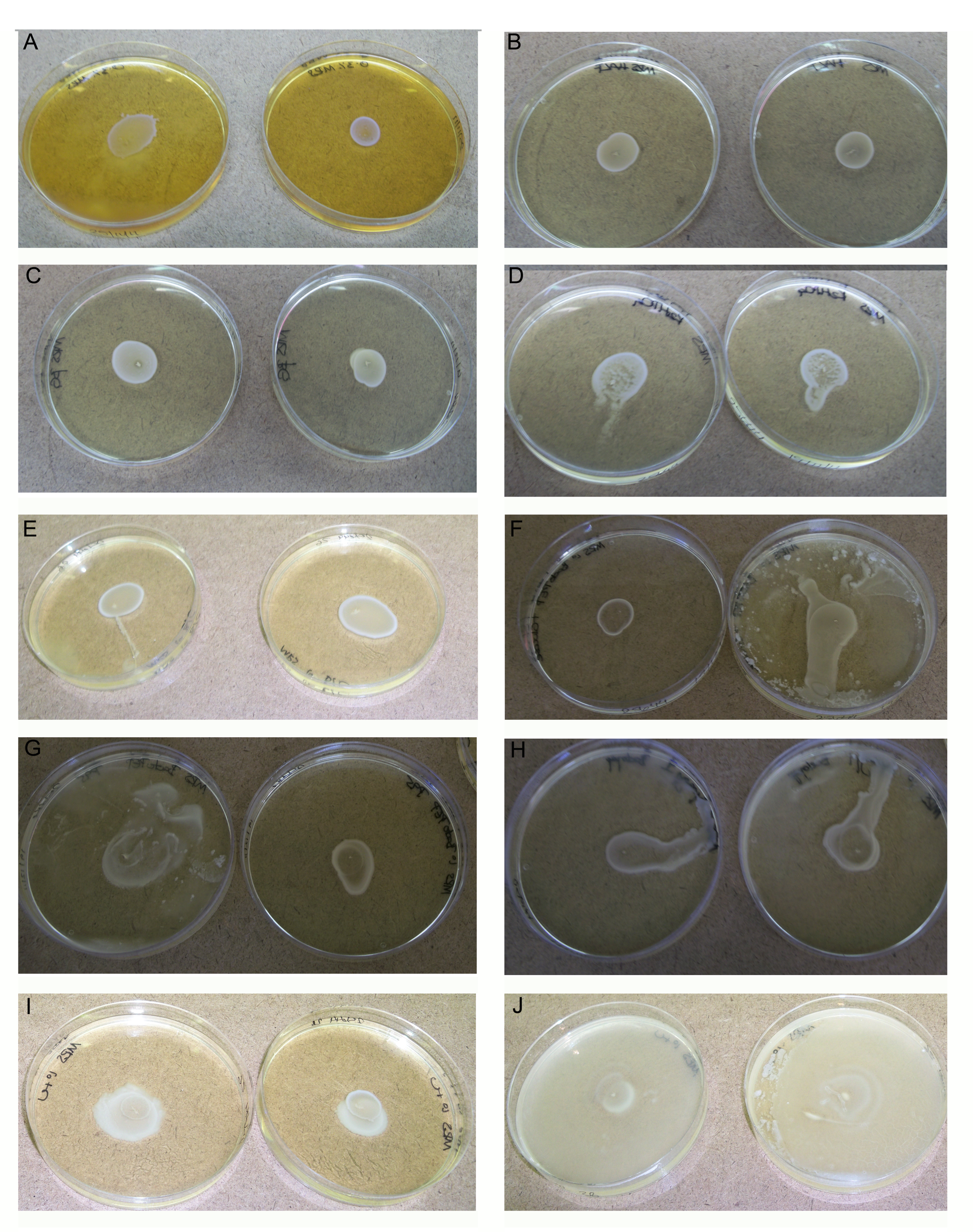

Supplement: Figure S6 — Failure of altered growth media to impart motility to L. ruminis ATCC25644 on semi-solid agar plates. A, B: Standard (A) or half-strength (B) MRS (Oxoid) inoculated with ATCC25644. C, D: MRS prepared from first principles with alternative phosphate sources, β-glycerophosphate (C) or K2HPO4 (D) and inoculated with ATCC25644. E, F: MRS prepared from first principles with alternative protein sources, Bactocasitone (E) and Bactopeptone (F) and inoculated with ATCC25644. G, H: MRS prepared from first principles with alternative carbohydrate sources, P95 (G) and Synergy I (H) and inoculated with ATCC25644. I, J: MRS prepared from first principles with uracil (0.005 g/500 ml) inoculated with ATCC25644 (I) or ATCC27782 (J). These photographs are of motility plates that were inoculated on different days. L. ruminis ATCC25644 is not motile under any of these conditions. L. ruminis ATCC27782 (J) is representative of a motile culture on semi-solid agar, and can be seen to cover the entire plate surface. (TIF) [file pone.0040592.s006.tif]
