# Supplementary material for: Characterization of Pro-Inflammatory Flagellin Proteins Produced by Lactobacillus ruminis and Related Motile Lactobacilli
Source: PLoS One. 2012 Jul 10;7(7):e40592. doi: 10.1371/journal.pone.0040592 (PMC3393694; doi:10.1371/journal.pone.0040592)
Supplement: Figure S3 — 16S rRNA gene tree and motility protein based phylogenetic trees. Trees were constructed using PHYML. Bootstrap values are given at each node. (PDF) [file pone.0040592.s003.pdf]

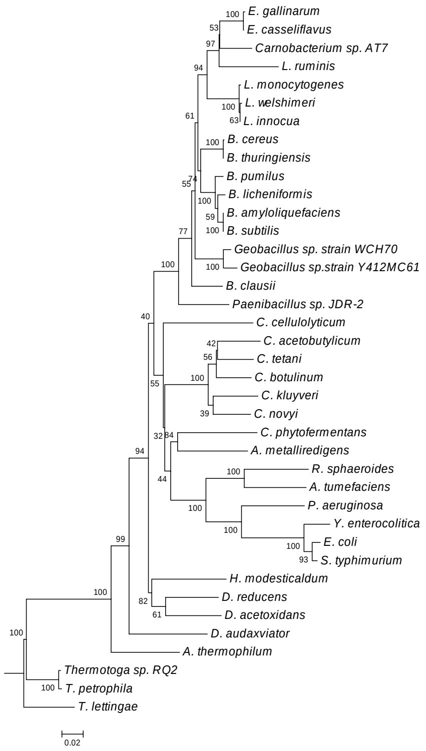

16S rRNA

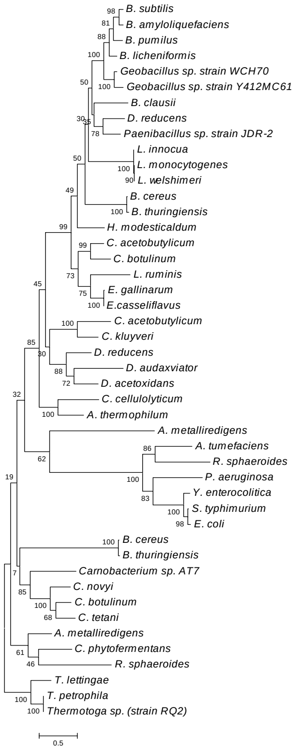

MotA

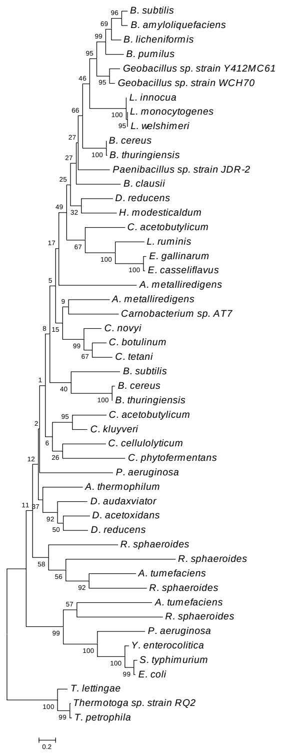

MotB

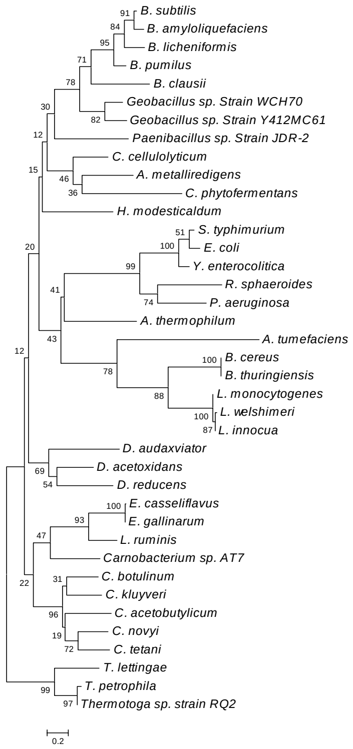

FlgB

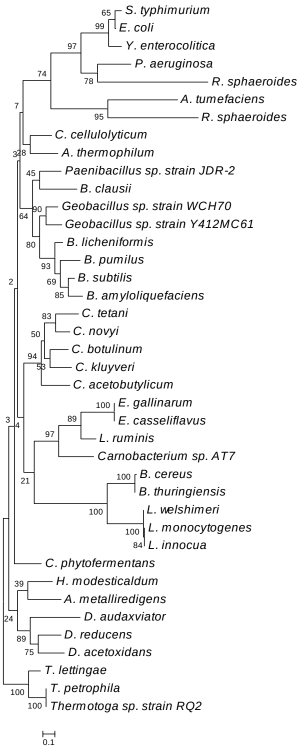

FlgC

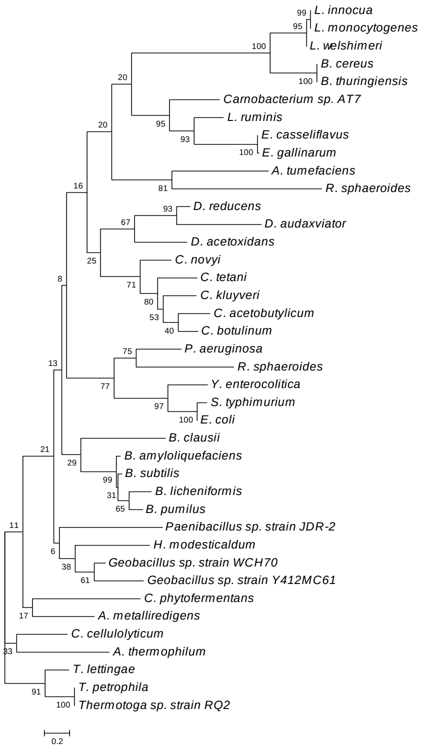

FliE

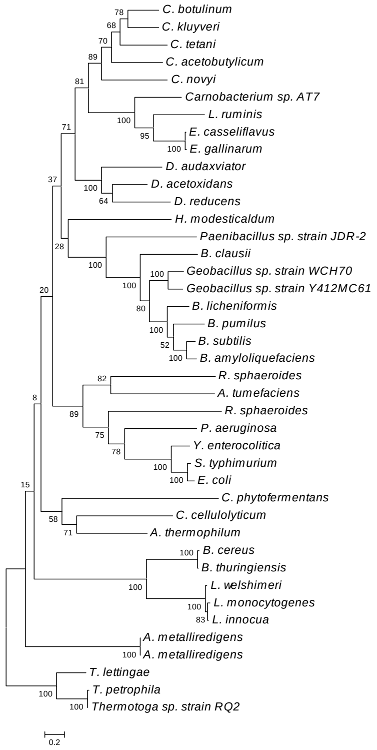

FliF

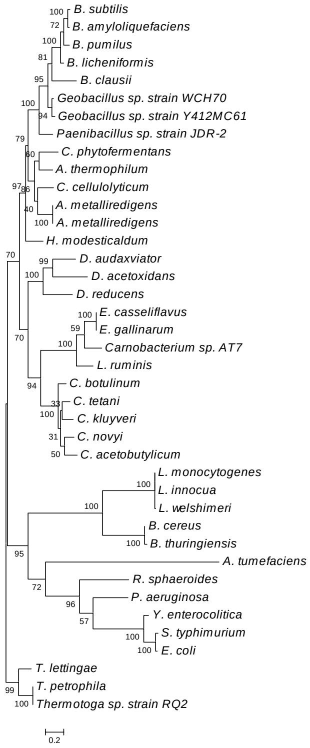

FliG

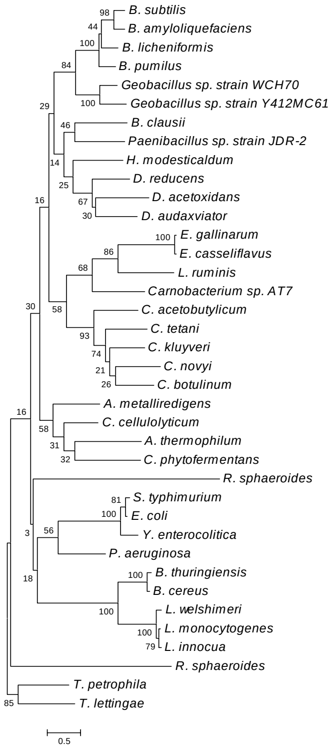

FliH

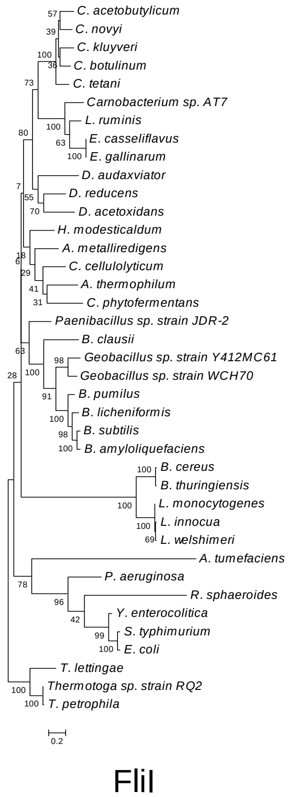

FliI

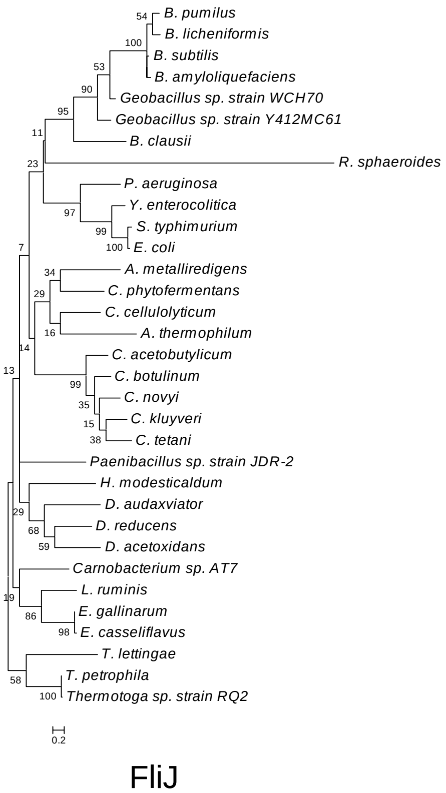

FliJ

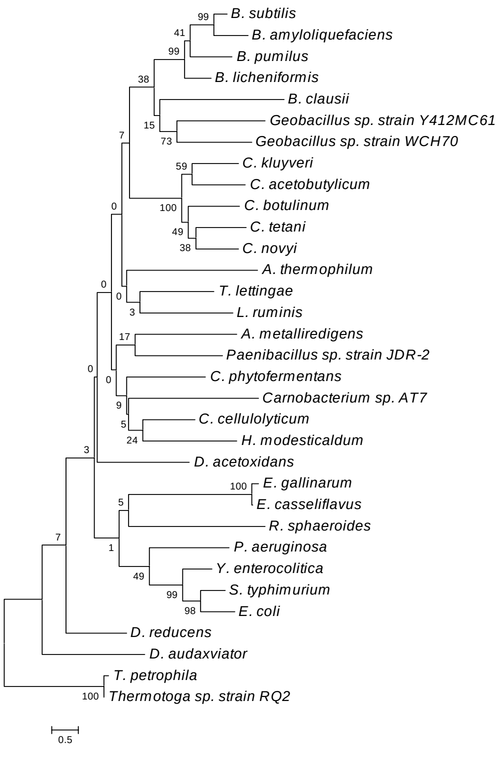

FliK

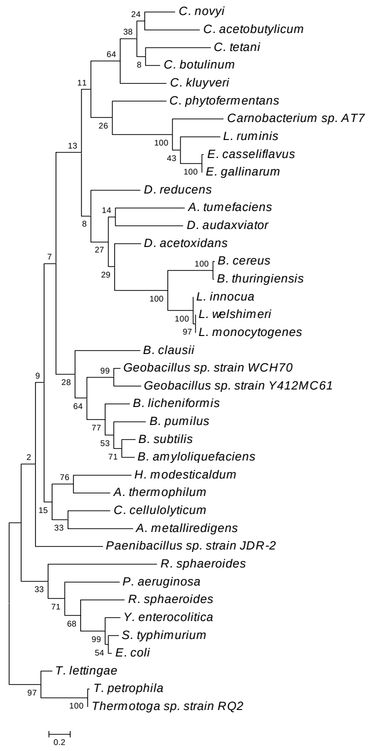

FlgD

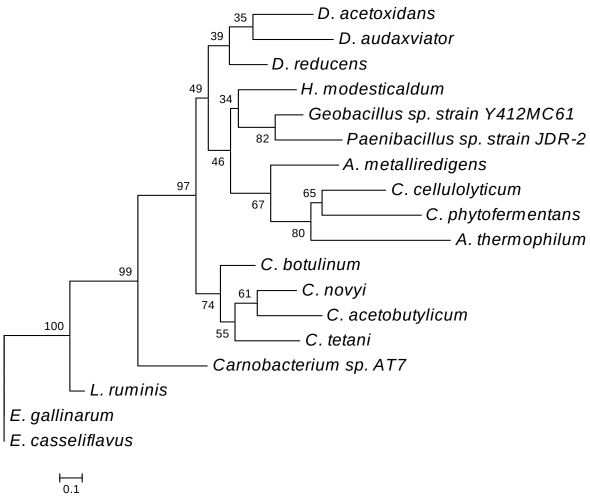

Flagellar Operon Protein

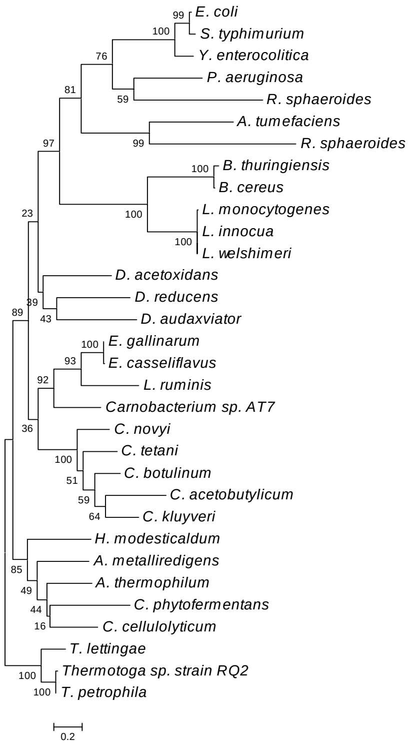

FlgE

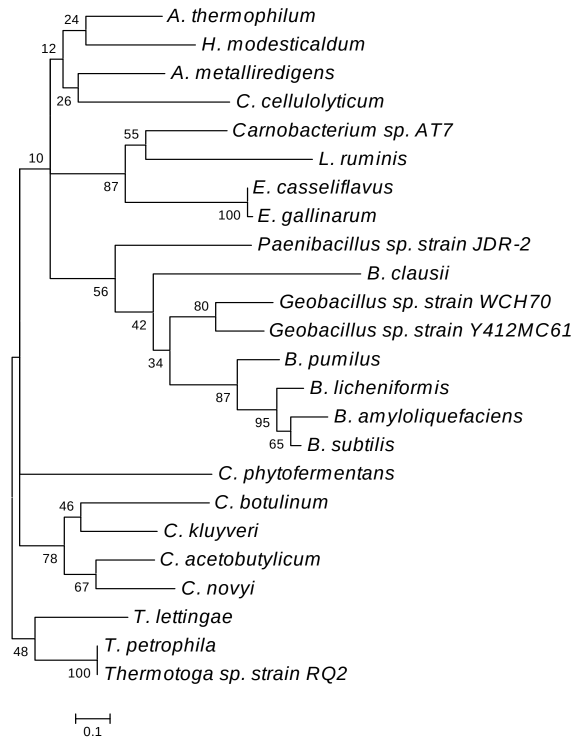

FlbD

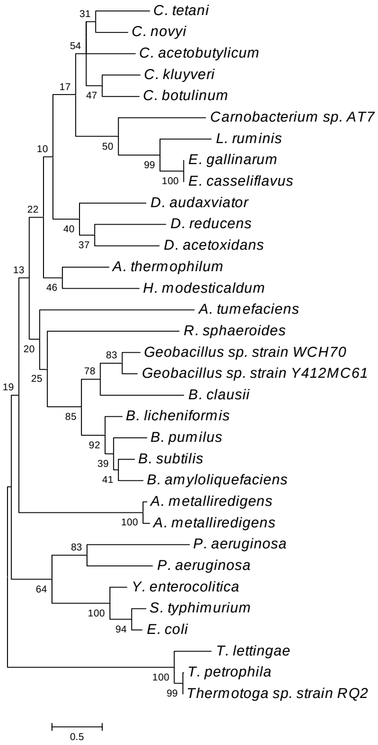

FliL

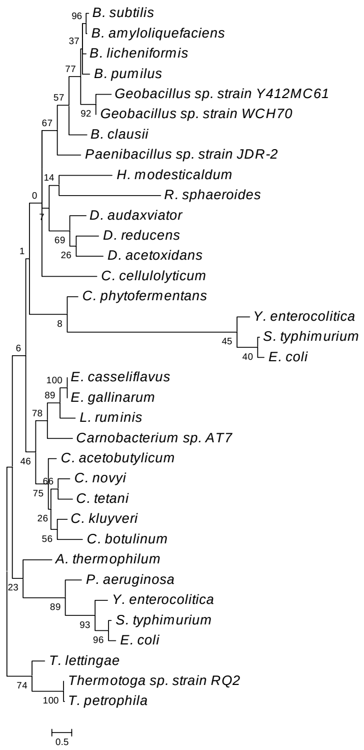

FliO/Z

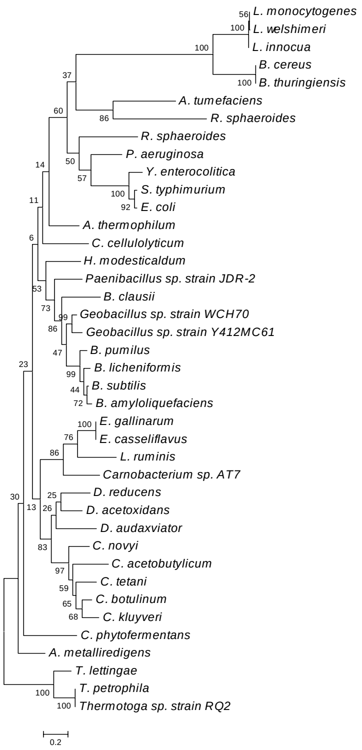

FliP

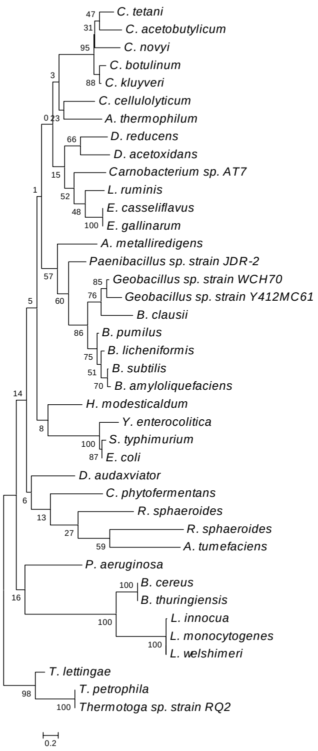

FliQ

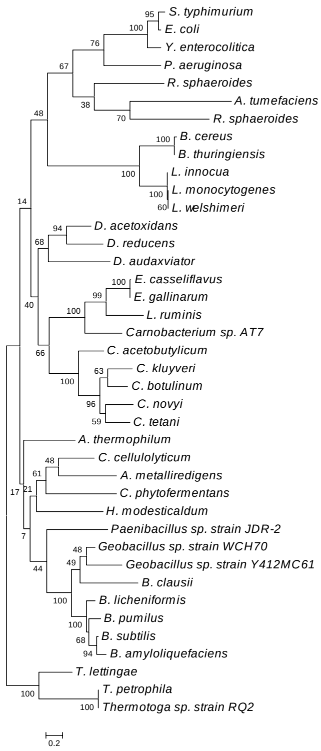

FliR

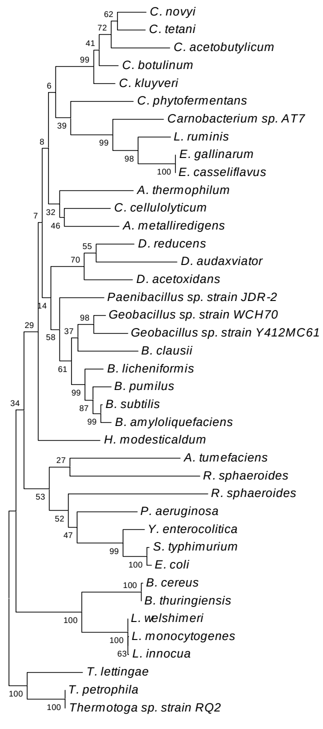

FlhB

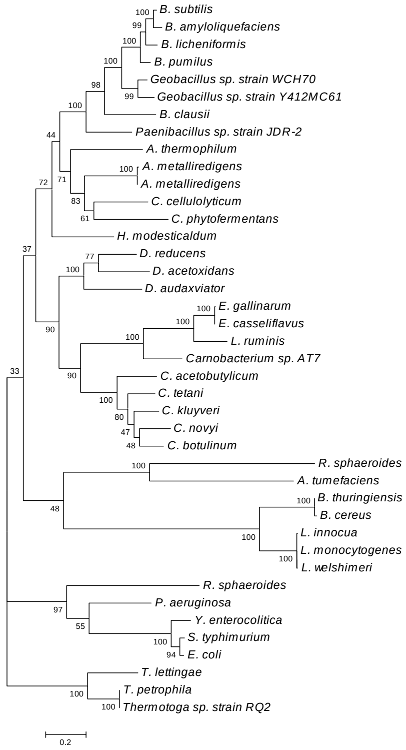

FlhA

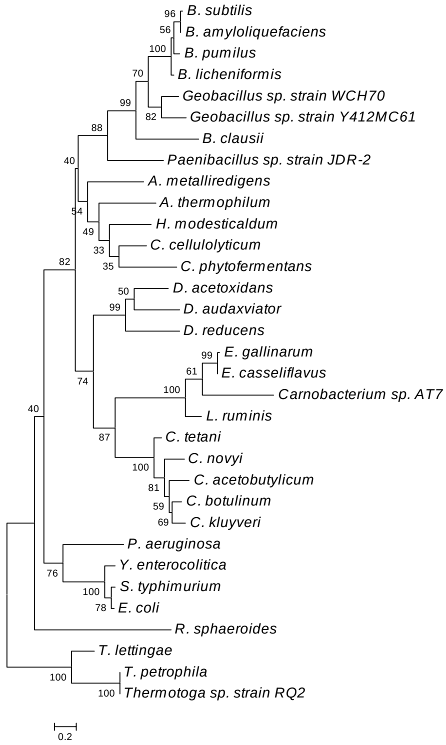

FliA

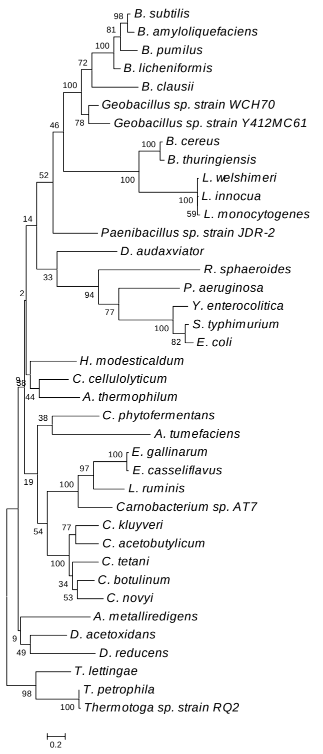

FlgF

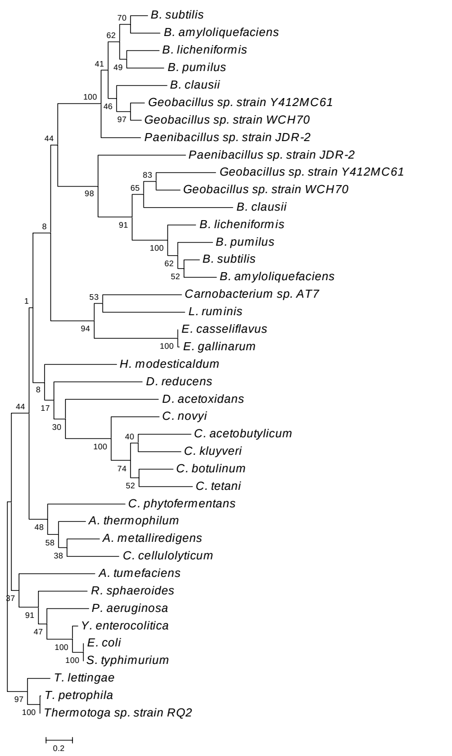

FlgG

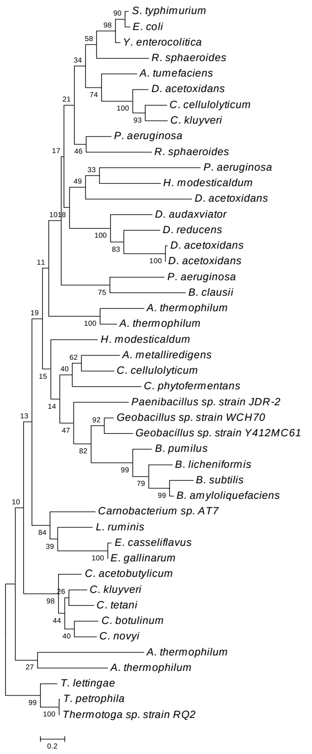

CheB

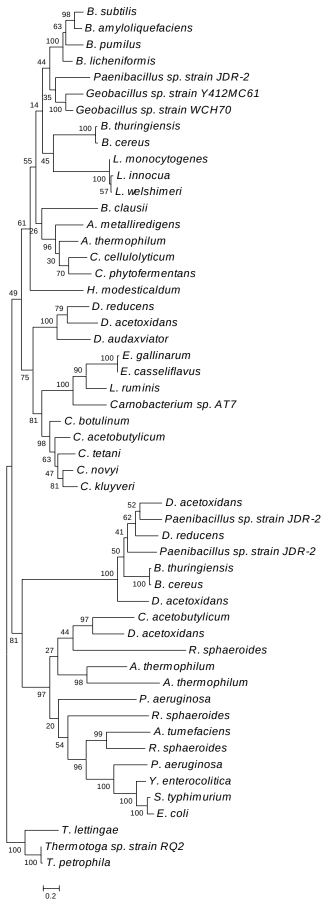

CheR

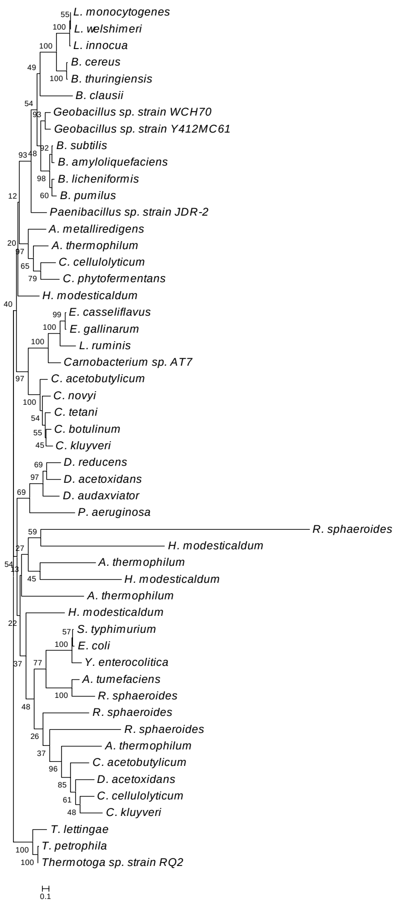

CheA

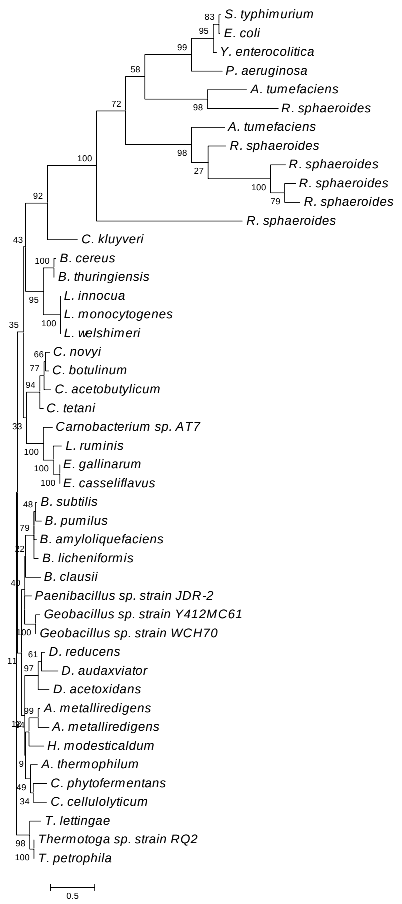

CheY

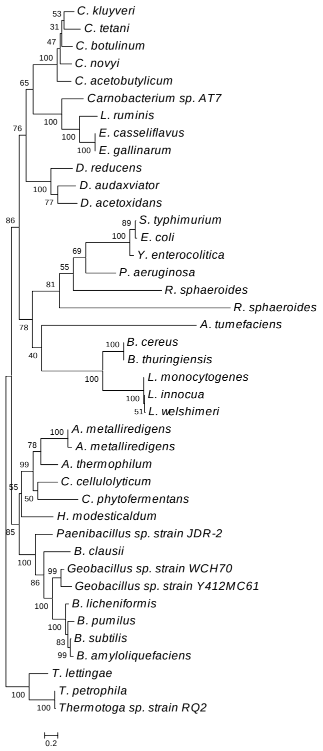

FliM

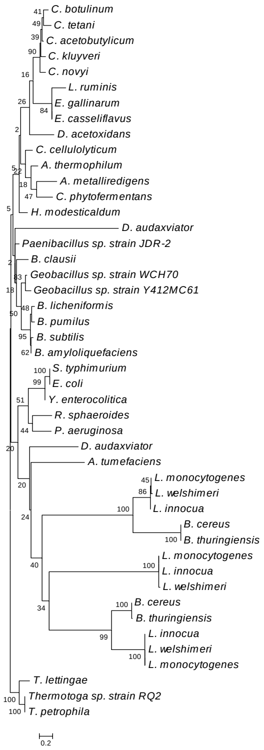

FliN/Y

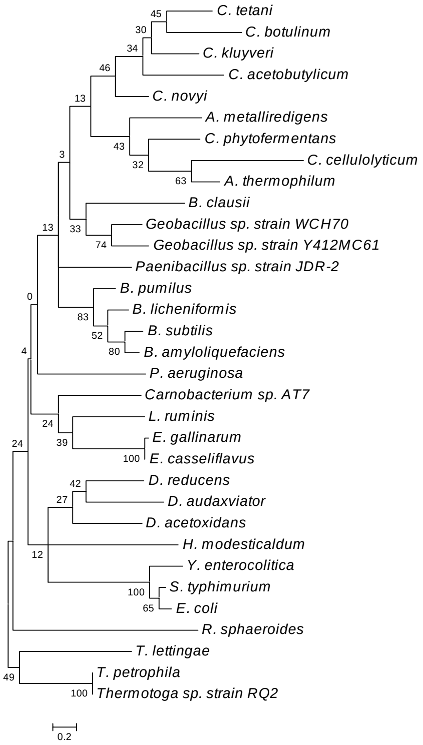

FlgM

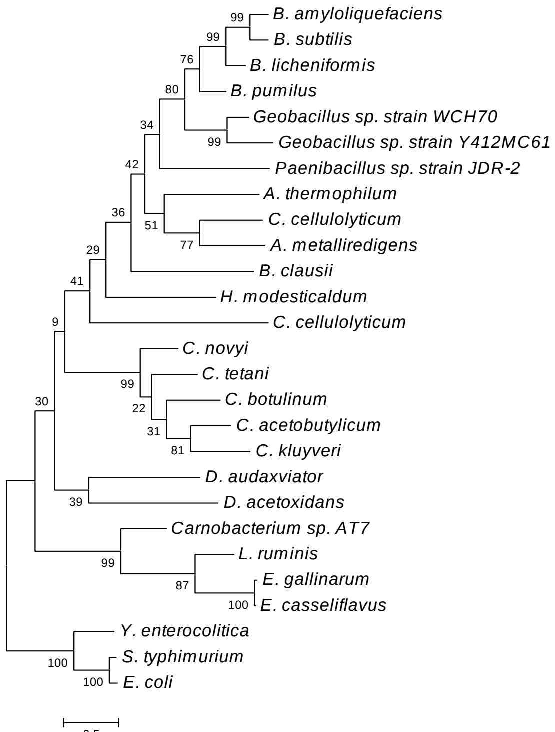

FlgN

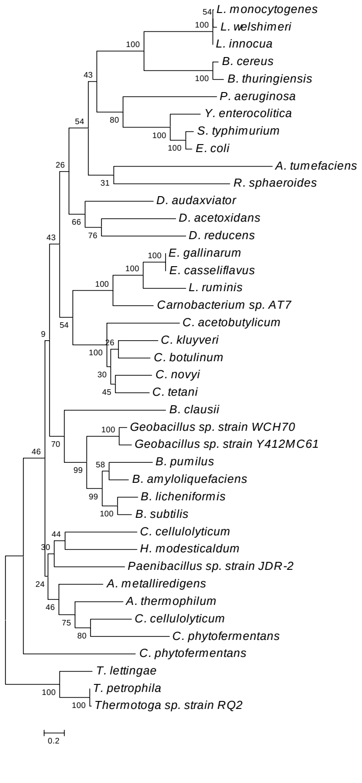

FlgK

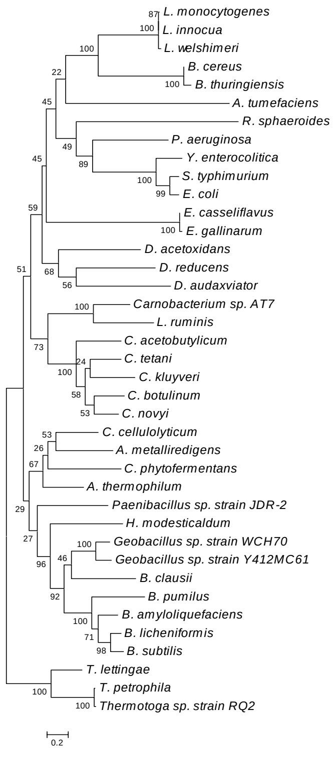

FlgL

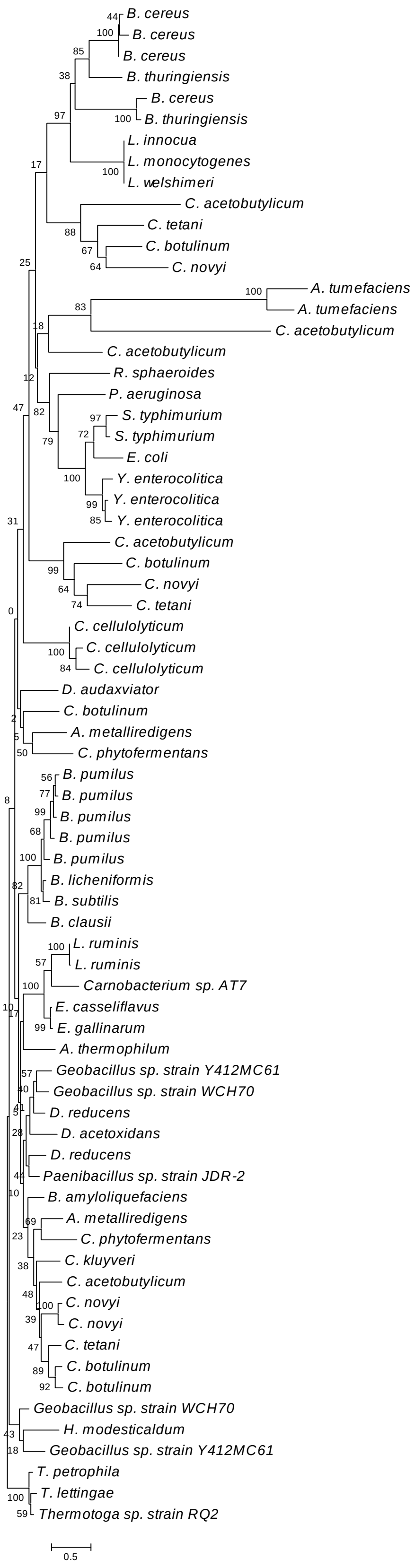

FliC

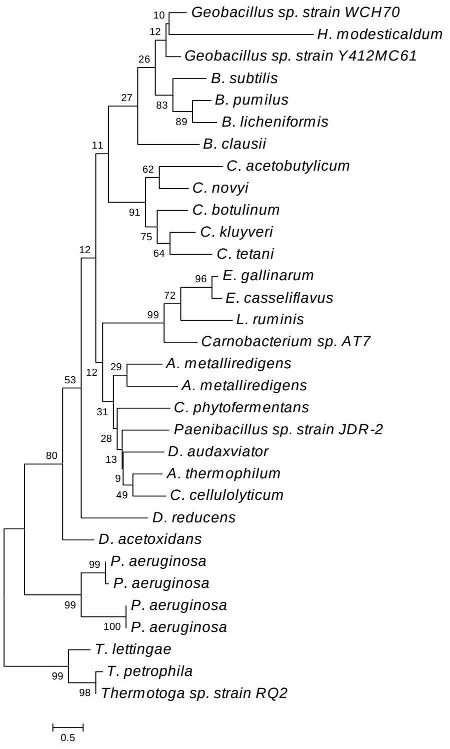

FlaG

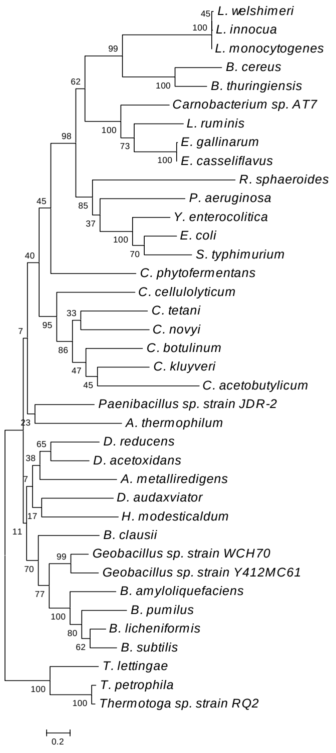

FliD

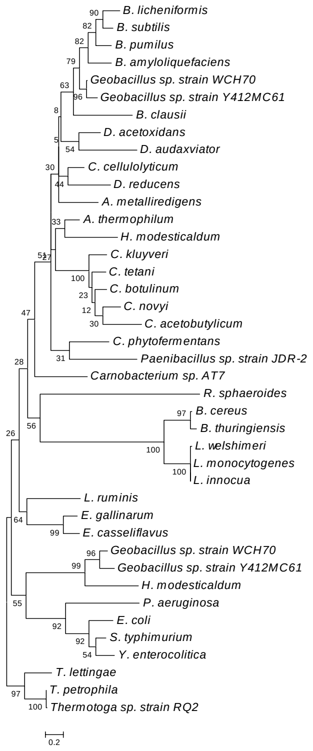

FliS
