# Supplementary material for: Characterization of Pro-Inflammatory Flagellin Proteins Produced by Lactobacillus ruminis and Related Motile Lactobacilli
Source: PLoS One. 2012 Jul 10;7(7):e40592. doi: 10.1371/journal.pone.0040592 (PMC3393694; doi:10.1371/journal.pone.0040592)
Supplement: Table S1 — Origin and phylogeny of motile Lactobacillus species described to date. (DOC) [file pone.0040592.s007.doc]

Table S1: Origin and phylogeny of motile *Lactobacillus* species described to date.

| **Year of isolation** | **Species** | **Type strain** | **Source** | **Clade ^A^** | **Reference to species motility** |
| --- | --- | --- | --- | --- | --- |
| 1970 | *L. mali* | DSM20444 | Wine must, cider | *L. salivarius* | [48] |
| 1973 | *L. ruminis* | ATCC27780 | Mammalian faeces, Bovine rumen | *L. salivarius* | [42] |
| 1982 | *L. agilis* | DSM20509 | Municipal sewage | *L. salivarius* | [85] |
| 2000 | *L. nagelii* | ATCC700692 | Partly fermented grape juice | *L. salivarius* | [86] |
| 2005 | *L. satsumensis* | DSM16230 | Mashes of shochu | *L. salivarius* | [87] |
| 2006 | *L. vini* | DSM20605 | Fermenting grape musts | *L. salivarius* | [73] |
| 2007 | *L. ghanensis* | DSM18630 | Fermenting cocoa | *L. salivarius* | [88] |
| 2008 | *L. capillatus* | DSM19910 | Fermented stinky tofu brine | *L. salivarius* | [89] |
| 2008 | *L. uvarum* | DSM19971 | Grape musts | *L. salivarius* | [90] |
| 2009 | *L. oeni* | DSM19972 | Bobal grape wines | *L. salivarius* | [91] |
| 2009 | *L. aquaticus* | DSM21051 | Korean freshwater pond | *L. salivarius* | [92] |
| 2009 | *L. sucicola* | DSM21376 | Sap of oak tree | *L. salivarius* | [92, 93] |

A. According to phylogeny of Felis et al., 2007 [22] .

85. Weiss N, Schillinger U, Laterneser M, Kandler O (1981*) Lactobacillus sharpeae* sp. nov and *Lactobacillus agilis* sp. nov., two new species of homofermentative, meso-diaminopimelic acid containing lactobacilli isolated from sewage. Zentralbl Bakteriol Mikrobiol Hyg l Abt C 2: 242-253.

86. Edwards CG, Collins MD, Lawson PA, Rodriguez AV (2000) *Lactobacillus nagelii* sp. nov., an organism isolated from a partially fermented wine. Int J Syst Evol Microbiol 50 699-702.

87. Endo A, Okada S (2005) *Lactobacillus satsumensis* sp. nov., isolated from mashes of shochu, a traditional Japanese distilled spirit made from fermented rice and other starchy materials. Int J Syst Evol Microbiol 55: 83-85.

88. Nielsen DS, Schillinger U, Franz CM, Bresciani J, Amoa-Awua W, et al. (2007) *Lactobacillus ghanensis* sp. nov., a motile lactic acid bacterium isolated from Ghanaian cocoa fermentations. Int J Syst Evol Microbiol 57: 1468-1472.

89. Chao SH, Tomii Y, Sasamoto M, Fujimoto J, Tsai YC, et al. (2008) *Lactobacillus capillatus* sp. nov., a motile bacterium isolated from stinky tofu brine. Int J Syst Evol Microbiol 58: 2555-2559.

90. Manes-Lazaro R, Ferrer S, Rossello-Mora R, Pardo I (2008) *Lactobacillus uvarum* sp. nov.--a new lactic acid bacterium isolated from Spanish Bobal grape must. Syst Appl Microbiol 31: 425-433.

91. Manes-Lazaro R, Ferrer S, Rossello-Mora R, Pardo I (2009) *Lactobacillus oeni* sp. nov., from wine. Int J Syst Evol Microbiol 59: 2010-2014.

92. Manes-Lazaro R, Song J, Pardo I, Cho JC, Ferrer S (2009) *Lactobacillus aquaticus* sp. nov., isolated from a Korean freshwater pond. Int J Syst Evol Microbiol 59: 2215-2218.

93. Irisawa T, Okada S (2009) *Lactobacillus sucicola* sp. nov., a motile lactic acid bacterium isolated from oak tree (Quercus sp.) sap. Int J Syst Evol Microbiol 59: 2662-2665.
