# Supplementary material for: Characterization of Pro-Inflammatory Flagellin Proteins Produced by Lactobacillus ruminis and Related Motile Lactobacilli
Source: PLoS One. 2012 Jul 10;7(7):e40592. doi: 10.1371/journal.pone.0040592 (PMC3393694; doi:10.1371/journal.pone.0040592)
Supplement: Table S2 — Lactobacillus strains and species used in this study. (DOC) [file pone.0040592.s008.doc]

Table S2: *Lactobacillus* strains and species used in this study

| **Species** | **Strain** | **Origin** | **Motility ^A^** | **Motility genes ^B^** |
| --- | --- | --- | --- | --- |
| *L. ruminis* | ATCC 25644 | Human | - | + |
| *L. ruminis* | ATCC 27780^T^ | Bovine rumen | + | + |
| *L. ruminis* | ATCC 27781 | Bovine rumen | + | + |
| *L. ruminis* | ATCC 27782 | Bovine rumen | + | + |
| *L. ruminis* | L5 | Human | - | + |
| *L. ruminis* | S21 | Human | - | + |
| *L. ruminis* | S23 | Human | - | + |
| *L. ruminis* | S36 | Human | - | + |
| *L. ruminis* | S38 | Human | - | + |
| *L. ghanensis* | L489^T^ | Cocoa bean fermentations | + | + |
| *L. mali* | DSM20444^T^ | Apple juice from cider press | + | + |
| *L. nagelii* | DSM13675^T^ | Partially fermented wine | + | + |

A, B: Data from this study.
