# Supplementary material for: Characterization of Pro-Inflammatory Flagellin Proteins Produced by Lactobacillus ruminis and Related Motile Lactobacilli
Source: PLoS One. 2012 Jul 10;7(7):e40592. doi: 10.1371/journal.pone.0040592 (PMC3393694; doi:10.1371/journal.pone.0040592)
Supplement: Table S3 — Closest homologs of L. ruminis motility proteins. (DOC) [file pone.0040592.s009.doc]

Table S3: Closest homologs* of *L. ruminis* motility proteins

| **Annotation** | **Locus Tag** | **Top non *L. ruminis* BLAST hit*** | **BLASTp top hit, excluding motile lactobacilli, ATCC27782 Query** | **% Identity ATCC27782** |
| --- | --- | --- | --- | --- |
| MCP | LRC_16170 | Methyl-accepting chemotaxis sensory transducer | *Enterococcus casseliflavus EC30,* ZP_05646024.1 | 37% |
| *motA* | LRC_16150 | MotA | *Enterococcus casseliflavus* ATCC12755*,* ZP_08144203.1 | 52% |
| *motB* | LRC_16140 | MotB | *Enterococcus casseliflavus* EC20, ZP_05655638.1 | 45% |
| Hypothetical protein | LRC_16130 | - | *-* | - |
| *flgB* | LRC_16120 | FlgB | *Enterococcus saccharolyticus* 30_1, ZP_09112718.1 | 56% |
| *flgC* | LRC_16110 | FlgC | *Enterococcus casseliflavus* ATCC12755, ZP_08144200.1 | 65% |
| *fliE* | LRC_16100 | Predicted protein | *Enterococcus gallinarum* EG2, ZP_05650941.1 | 51% |
| *fliF* | LRC_16090 | FliF | *Enterococcus gallinarum* EG2, ZP_05650942.1 | 44% |
| *fliG* | LRC_16080 | FliG | *Enterococcus gallinarum EG2,* ZP_05650943.1 | 69% |
| *fliH* | LRC_16070 | Predicted protein | *Enterococcus casseliflavus* EC30, ZP_05646033.1 | 20% |
| *fliI* | LRC_16060 | Flagellum specific ATP synthase | *Enterococcus casseliflavus* ATCC12755, ZP_08144195.1 | 77% |
| *fliJ* | LRC_16050 | Putative flagellar protein | *Enterococcus casseliflavus* ATCC12755, ZP_08144194.1 | 44% |
| *fliK* | LRC_16040 | Hypothetical protein | *Enterococcus saccharolyticus* 30_1, ZP_09112710.1 | 44% |
| *flgD* | LRC_16030 | Predicted protein | *Enterococcus gallinarum* EG2, ZP_05650948.1 | 43% |
| Flagellar operon protein | LRC_16020 | Predicted protein | *Enterococcus casseliflavus* EC30, ZP_05646038.1 | 58% |
| *flgE* | LRC_16010 | Flagellar hook protein | *Enterococcus casseliflavus* EC30, ZP_05646039.1 | 56% |
| *flbD* | LRC_16000 | Putative flagellar protein | *Carnobacterium* sp. 17-4, YP_004375661.1 | 51% |
| *fliL* | LRC_15990 | Hypothetical protein | *Enterococcus casseliflavus* ATCC12755, ZP_08144188.1 | 45% |
| *fliO* | LRC_15980 | FliZ | *Enterococcus casseliflavus* ATCC12755, ZP_08144187.1 | 40% |
| *fliP* | LRC_15970 | FliP | *Enterococcus casseliflavus* ATCC12755, ZP_08144186.1 | 60% |
| *fliQ* | LRC_15960 | Flagellar biosynthesis protein | *Enterococcus gallinarum EG2, ZP_05650955.1* | 69% |
| *fliR* | LRC_15950 | FliR | *Enterococcus casseliflavus* EC30, ZP_05646045.1 | 42% |
| *flhB* | LRC_15940 | FlhB | *Enterococcus casseliflavus* EC20, ZP_05655659.1 | 50% |
| *flhA* | LRC_15930 | FlhA | *Enterococcus casseliflavus* EC20, ZP_05655660.1 | 66% |
| *fliA* | LRC_15920 | RNA polymerase sigma factor *whiG* | *Enterococcus casseliflavus* EC30, ZP_05646048.1 | 65% |
| *flgF* | LRC_15910 | Flagellar hook-basal body complex protein | *Enterococcus casseliflavus* EC30, ZP_05646049.1 | 54% |
| *flgG* | LRC_15900 | FlgG | *Enterococcus casseliflavus* EC30, ZP_05646050.1 | 41% |
| ftsE-like | LRC_15890 | FtsE | *Enterococcus casseliflavus* EC20, ZP_05655664.1 | 51% |
| MCP | LRC_15880 | MCP | *Enterococcus gallinarum* EG2, ZP_05650963.1 | 38% |
| *cheW* | LRC_15870 | Predicted protein | *Enterococcus casseliflavus* EC30, ZP_05646053.1 | 46% |
| *cheD* | LRC_15860 | CheD | *Enterococcus casseliflavus* EC30, ZP_05646054.1 | 59% |
| *cheB* | LRC_15850 | Chemotaxis response regulator protein-glutamate methylesterase | *Enterococcus casseliflavus* EC30, ZP_05646055.1 | 53% |
| *cheR* | LRC_15840 | Chemotaxis protein methyltransferase, CheR | *Enterococcus casseliflavus ATC*C12755, ZP_08144173.1 | 60% |
| *cheA* | LRC_15830 | Chemotaxis histidine kinase | *Enterococcus gallinarum* EG2, ZP_05650968.1 | 58% |
| *cheC* | LRC_15820 | Hypothetical protein | *Enterococcus saccharolyticus* 30_1, ZP_09112688.1 | 58% |
| *cheY* | LRC_15810 | Sporulation initiation phosphotransferase F | *Enterococcus casseliflavus* ATCC12755, ZP_08144169.1 | 83% |
| *cheW* | LRC_15800 | Hypothetical protein | *Enterococcus saccharolyticus* 30_1, ZP_09112686.1 | 56% |
| *fliM* | LRC_15790 | FliM | *Enterococcus casseliflavus* ATCC12755, ZP_08144167.1 | 63% |
| *fliY* | LRC_15780 | FliY | *Enterococcus gallinarum* ATCC12755, ZP_08144166.1 | 55% |
| *flgM* | LRC_15770 | Hypothetical protein | *Enterococcus casseliflavus* ATCC12755. ZP_08144165.1 | 41% |
| *flgN* | LRC_15760 | Predicted protein | *Enterococcus casseliflavus* EC30, ZP_05646064.1 | 46% |
| *flgK* | LRC_15750 | FlgK | *Enterococcus casseliflavus* EC20, ZP_05655678.1 | 51% |
| *flgL* | LRC_15740 | Flagellin | *Enterococcus casseliflavus* EC20, ZP_05655679.1 | 51% |
| Hypothetical protein | LRC_15730 | Hypothetical protein PECL_1514** | *Pediococcus claussenii* ATCC BAA-344, AEV95735.1 | 30% |
| Hypothetical protein | LRC_15720 | DNA-directed RNA polymerase specialized sigma subunit, sigma24 family protein | *Lactobacillus brevis subsp. gravesensis ATCC27305,* ZP_03938825.1 | 28% |
| Hypothetical protein | LRC_15710 | Enniatin synthetase** | *Fusarium equiseti,* Q00869.2 | 32% |
| *fliC*1 | LRC_15700 | Flagellin-domain containing protein | *Caldicellulosiruptor kristjanssonii* 177R1B. YP_004026781.1 | 62% |
| Glycosyl-transferase | LRC_15690 | Glycosyltransferase family 2 | *Clostridium sp.* DL-VIII, *ZP_09206896.1* | 33% |
| *fliC*2 | LRC_15680 | Flagellin-domain containing protein | *Mahella australiensis* 50-1 BON, YP_004463585.1 | 61% |
| Glycosyl-transferase | LRC_15670 | Hypothetical protein ANACOL_00882 | *Anaerotruncus colihominis* DSM 17241, ZP_02441601.1 | 32% |
| *flaG* | LRC_15660 | Predicted protein | *Enterococcus casseliflavus* EC30, ZP_05646068.1 | 34% |
| *fliD* | LRC_15650 | Flagellar hook-associated protein | *Enterococcus casseliflavus* EC30*,* ZP_05646069.1 | 43% |
| Hypothetical protein | LRC_15640 | Acetyltransferase 1 -ike** | *Ectocarpus siliculosus,* CBN77876.1 | 33% |
| *fliS* | LRC_15630 | FliS | *Enterococcus casseliflavus* EC30, ZP_05646071.1 | 44% |

* Homologs in motile *Lactobacillus* genomes, *L. ruminis*, *L. mali* and *L. acidipiscis,* were excluded from this table.

** BLAST hit had high E-value (>1.0).
