# Supplementary material for: Characterization of Pro-Inflammatory Flagellin Proteins Produced by Lactobacillus ruminis and Related Motile Lactobacilli
Source: PLoS One. 2012 Jul 10;7(7):e40592. doi: 10.1371/journal.pone.0040592 (PMC3393694; doi:10.1371/journal.pone.0040592)
Supplement: Table S4 — Expression analysis of flagellum biogenesis and chemotaxis genes in L. ruminis derived by type I microarray. Values tabulated are expression ratios of ATCC27782 relative to ATCC25644. Values in parentheses represent relative fold differences in expression of ATCC25644 relative to ATCC27782. Relative data for qRT-PCR are also shown. Genes with at least 5 fold relative difference in expression and P-values <1.0×10^−4^ are in bold-face. † Normalized to expression level of fliC2, ATCC27782. ‡ Microarray probes for fliC could not distinguish between fliC1 and fliC2, although qRT-PCR shows only fliC2 is expressed. ND = No data. (DOC) [file pone.0040592.s010.doc]

**Table S4: Expression analysis of flagellum biogenesis and chemotaxis genes in *L. ruminis* derived by type I microarray**.

|  | Motile growth phase | | Non-motile growth phase | |
| --- | --- | --- | --- | --- |
| Relative fold change in: | ATCC27782 | qRT-PCR | ATCC27782 | qRT-PCR |
| MCP | 1.3 |  | 1.7 |  |
| *motA* | **6.6** |  | 2.2 |  |
| *motB* | **21.6** |  | 4.2 |  |
| *flgB* | **16.2** |  | 4.3 |  |
| *flgC* | **12.1** |  | 3.5 |  |
| *fliE* | **12.9** |  | 2.5 |  |
| *fliF* | **14.1** |  | 2.7 |  |
| *fliG* | **13.2** |  | 3.1 |  |
| *fliH* | **13.7** |  | 2.0 |  |
| *fliI* | **12.9** |  | 3.2 |  |
| *fliJ* | **12.5** |  | 3.3 |  |
| *fliK* | **17.1** |  | 4.6 |  |
| *flgD* | **12.6** |  | 4.4 |  |
| Flagellar operon protein | **9.8** |  | 4.5 |  |
| *flgE* | **17.7** |  | 2.8 |  |
| *flbD* | **9.0** |  | 2.2 |  |
| *fliL* | **17.7** |  | 2.3 |  |
| *fliZ* | **22.3** |  | 2.1 |  |
| *fliP* | **41.6** |  | 2.6 |  |
| *fliQ* | **26.9** |  | 1.9 |  |
| *fliR* | **23.3** |  | 2.4 |  |
| *flhB* | **17.9** |  | 2.2 |  |
| *flhA* | **16.5** |  | 3.0 |  |
| *fliA* | **14.5** |  | 2.2 |  |
| *flgF* | **17.1** |  | 3.1 |  |
| *flgG* | **14.9** |  | 2.8 |  |
| *ftsE*-like | **7.2** |  | 1.9 |  |
| *MCP* | **34.6** |  | 4.5 |  |
| *cheW* | **27.9** |  | 2.8 |  |
| *cheD* | **32.8** |  | 3.1 |  |
| *cheB* | **21.8** |  | 3.4 |  |
| *cheR* | **25.0** |  | 2.1 |  |
| *cheA* | **29.8** |  | 4.0 |  |
| *cheC* | **24.8** |  | 2.6 |  |
| *cheY* | **38.2** |  | 3.4 |  |
| *cheW* | **35.6** |  | 3.3 |  |
| *fliM* | **19.5** | **160010.44** | 3.3 | **28487.54** |
| *fliY* | **15.2** |  | 4.7 |  |
| *flgM* | **16.9** |  | 2.8 |  |
| *flgN* | **34.2** |  | 2.7 |  |
| *flgK* | **21.3** |  | 3.7 |  |
| *flgL* | **28.5** |  | 2.2 |  |
| Hypothetical protein, (LRC_15730) | **(20.1)** | **(477.59)** | 0.5 | **(12.726)** |
| Hypothetical protein, (LRC_15720) | 1.5 |  | 2.4 |  |
| Hypothetical protein, (LRC_15710) | 0.4 |  | 0.6 |  |
| *fliC***^‡^** | **90.7** | **770.49** | **49.6** | **327.9** |
| *fliC1* LRC_15700 | ND | **0** | ND | ND |
| *fliC2* LRC_15680 | ND | **1^†^** | ND | ND |
| Possible glycosyl transferase | **31.0** |  | 3.5 |  |
| *flaG* | **25.9** |  | 4.3 |  |
| *fliD* | **27.4** |  | 3.4 |  |
| Hypothetical protein, (LRC_15640) | **44.8** |  | 3.6 |  |
| *fliS* | **12.7** |  | 3.1 |  |
| Sigma 70 like ECF sigma factor (LRC_04420/ANHS_51c) | **(800.1)** |  | **(12.5)** |  |

Values tabulated are expression ratios of ATCC27782 relative to ATCC25644. Values in parentheses represent relative fold differences in expression of ATCC25644 relative to ATCC27782. Relative data for qRT-PCR are also shown. Genes with at least 5 fold relative difference in expression and P-values < 1.0x10^-4^ are in bold-face. ^†^ Normalized to expression level of *fliC*2, ATCC27782. ^‡^ Microarray probes for *fliC* could not distinguish between *fliC1* and *fliC2*, although qRT-PCR shows only *fliC*2 is expressed. ND = No data.
