# Supplementary material for: Characterization of Pro-Inflammatory Flagellin Proteins Produced by Lactobacillus ruminis and Related Motile Lactobacilli
Source: PLoS One. 2012 Jul 10;7(7):e40592. doi: 10.1371/journal.pone.0040592 (PMC3393694; doi:10.1371/journal.pone.0040592)
Supplement: Table S5 — Primers used in this study. (DOC) [file pone.0040592.s011.doc]

Table S5: Primers used in this study.

| Primer Name | Primer Sequence |
| --- | --- |
| B-Actin_F | 5’-ATTGGCAATGAGCGGTTC-3’ |
| B-Actin_R | 5’-TGAAGGTAGTTTCGTGGATGC-3’; |
| TLR5_F | 5’- TCTCCACAGTCACCAAACCA-3’; |
| TLR5_R | 5’-AAGCTGGGCAACTATAAGGTCA-3’; |
|  |  |
| ANHS_515c FliC1_SmaI_F | 5’-GCGCGCCCCGGGTGCGTATTAACACAAACGTCGC-3’ |
| ANHS_515c FliC1_XhoI_R | 5’-GCGCGCCTCGAGTTAGCCTTGAAGCAAGTTCA-3’ |
|  |  |
| *groEL*_F | 5’-CGACGATTACGAATGACGGC-3’ |
| *groEL*_R | 5’-AGTTGCCGTTGTCGTTCCGT-3’ |
| *fliM*_F | 5’-CGATGCGCCTAAAGTCAGAG-3’ |
| *fliM*_R | 5’-CACATTCGTCCTGAGCTGAG-3’ |
| LRC_15730_F | 5’-CATGCTCGTTGAAGCTGACC-3’ |
| LRC_15730_R | 5’-CACGTAGCTCAAAGGAGCGA-3’ |
|  |  |
| *fliC* 1_LRC_15700-F | 5’-AAATCGGTGGTTTGACACAG-3’ |
| *fliC* 1_LRC_15700-R: | 5’-GAACCACGATCTGTTTCT-3’; |
| *fliC2*_LRC_15680-F: | 5’-AAATCGGTGGTTTGACACAA-3’; |
| *fliC* 2_LRC_15680-R | 5’-CCACGATCCTCTGCC-3’; |
| *era*_F: | 5’-TAAACCGCAAAGCCGTCTGG-3’; |
| *era*_R: | 5’-ATGAAATCATCGCCGCGGCC-3’ |
|  |  |
| SP1_ANHS_518 | 5’-GATTAGGCGCTTGGTCTGTT-3’; |
| SP2_ ANHS_518 | 5'-CATTTGGTCTGAAGTTCAGG-3' |
| SP3_ ANHS_518 | 5'-CATGTCGCATCAAATGCTTG-3' |
| SP1_flagellin | 5’-AACGTTGCATCTGTCGATCC-3’ |
| SP2_flagellin | 5'-GATGGAGCCACGATCTGTTT-3' |
| SP3_flagellin: | 5'-CACCGATTTGTGACTTCATC-3'; |
| SP1_AHS51c: | 5’-CGTCGTTGTCTGATTGTAGC-3’; |
| SP2_ANHS_51c: | 5 '-GCCTCTTGAAGCAAATCGTC-3'; |
| SP3_ANHS_51c: | 5'-CCTAATAACCAACGGCTTGT-3' |
|  |  |
| 1054561:1055463F | 5’-CGAAAGGTTCCTTCAGTTGC-3’ |
| 1054561:1055463R | 5’-GCAGCCAGGTACAGCTCATA-3’ |
|  |  |
| 653884:654800F: | 5’-TTTCATCGTTATGCCAGCAG-3’ |
| 653884:654800R: | 5’-CACGTTTCACCTGATCGAAAT-3’ |
|  |  |
| 626252:627921F: | 5’-AATCACACGTCCGACAAACA-3’ |
| 626252:627921R | 5’-TTGCTTCAATCCAAGGCTCT-3’ |
|  |  |
| 380111:380820F | 5’-GGAAGGAAAACGTGTTTCAAAT-3’ |
| 380111:380820R: | 5’-CAATTCCACGCATTGTTACG-3’ |
|  |  |
| 1654861:1656335F: | 5’-GCGTTGTGAGACATTGGTTG-3’ |
| 1654861:1656335R | 5’-TCCTCTTGGCACAGCTTCTC-3’ |
|  |  |
| 1338430:1339559F | 5’-GATTCCAGCGTGGAAATGAT-3’ |
| 1338430:1339559R | 5’-TGCACTCTTCATCGGTTTTG-3’ |
|  |  |
| 644444:645201F: | 5’-ACGAACTGTCGGAAAACGAC-3’ |
| 644444:645201R: | 5’-CGACATCTTATCGCCAGGTT-3’ |
|  |  |
| 27F | 5’-AGAGTTTGATCMTGGCTCAG-3’ |
| 1492R | 5’-TACGGCACCTTGTTACGACTT-3’ |
