# Supplementary material for: Characterization of Pro-Inflammatory Flagellin Proteins Produced by Lactobacillus ruminis and Related Motile Lactobacilli
Source: PLoS One. 2012 Jul 10;7(7):e40592. doi: 10.1371/journal.pone.0040592 (PMC3393694; doi:10.1371/journal.pone.0040592)
Supplement: Table S6 — Uniprot and NCBI accession numbers for motility proteins used for phylogenetic analysis. (DOC) [file pone.0040592.s012.doc]

|  | **MotA** | **MotB** | **FlgB** | **FlgC** | **FliE** | **FliF** | **FliG** | **FliH** |
| --- | --- | --- | --- | --- | --- | --- | --- | --- |
| *A. tumefaciens* | Q44456 | Q7CT75/A9CK71 | Q44335 | Q44336 | Q44337 | Q7D1A2 | Q44458 |  |
| *A. metalliredigens* | A6TK10/A6TRP8 | A6TK11/A6TRP7 | A6TRR2 | A6TRR1 | A6TRR0 | A6TRQ9/A6TKW7 | A6TRQ8/A6TKW8 | A6TRQ7 |
| *A. thermophilum* | B9MR69 | B9MR70 | B9MM28 | B9MM27 | B9MM26 | B9MM25 | B9MM24 | B9MM23 |
| *B. amyloliquefaciens* | A7Z3Y2 | A7Z3Y1 | A7Z4N8 | A7Z4N9 | A7Z4P0 | A7Z4P1 | A7Z4P2 | A7Z4P3 |
| *B. cereus* | Q817M5/Q81FG5 | Q817M6/Q81FG4 | Q81FE9 | Q81FE8 | Q81FE7 | Q81FE6 | Q81FE5 | Q81FE4 |
| *B. thuringiensis* | Q6HD12/Q6HKS4 | Q6HD13/Q6HKS3 | Q6HKQ8 | Q6HKQ7 | Q6HKQ6 | Q6HKQ5 | Q6HKQ4 | Q6HKQ3 |
| *B. licheniformis* | Q65KI9 | Q65KJ0 | Q65JN1 | Q65JN0 | Q65JM9 | Q65JM8 | Q65JM7 | Q65JM6 |
| *B. pumilus* | A8FCH6 | A8FCH5 | A8FD82 | A8FD83 | A8FD84 | A8FD85 | A8FD86 | A8FD87 |
| *B. clausii* | Q5WGI7 | Q5WGI6 | Q5WFQ3 | Q5WFQ4 | Q5WFQ5 | Q5WFQ6 | Q5WFQ7 | Q5WFQ8 |
| *B. subtilis* | P2861 | P28612/P39064 | P24500 | P24501 | P24502 | P23447 | P23448 | P23449 |
| *Carnobacterium sp. AT7* | A8X5W8 | A8X5W7 | A8X5Y5 | A8X5Y4 | A8X5Y2 | A8X5Y1 | A8X5Y0 | A8X5X9 |
| *C. acetobutylicum* | Q97I10/Q97M94 | Q97I11/Q97M93 | Q97H48 | Q97H49 | Q97H50 | Q97H51 | Q97H52 | Q97H53 |
| *C. botulinum* | C3KZ03/C3L1E0 | C3L1D9 | C3L1T9 | C3L1T8 | C3L1T7 | C3L1F0 | C3L1E9 | C3L1E8 |
| *C. cellulolyticum* | B8I571 | B8I570 | B8I3Q7 | B8I3Q6 | B8I3Q5 | B8I3Q4 | B8I3Q3 | B8I3Q2 |
| *C. kluyveri* | A5N3S8 | A5N3S7 | A5N7B1 | A5N7B2 | A5N7B3 | A5N7B4 | A5N7B5 | A5N7B6 |
| *C. novyi* | A0Q033 | A0Q034 | A0Q020 | A0Q021 | A0Q022 | A0Q023 | A0Q024 | A0Q025 |
| *C. phytofermentans* | A9KNF0 | A9KNE9 | A9KNG4 | A9KNG3 | A9KNG2 | A9KNG1 | A9KNG0 | A9KNF9 |
| *C. tetani* | Q893Y9 | Q893Z0 | Q893X8 | Q893X9 | NT02CT1784 | Q893Y0 | Q893Y1 | Q893Y2 |
| *D. audaxviator* | B1I5I2 | B1I5I3 | B1I5F4 | B1I5F3 | B1I5F2 | B1I5F1 | B1I5F0 | B1I5E9 |
| *D. acetoxidans* | C8W1C4 | C8W1C5 | C8W1F4 | C8W1F5 | C8W1F6 | C8W1F7 | C8W1F8 | C8W1F9 |
| *D. reducens* | A4J1F0/A4J7A0 | A4J1E9/A4J799 | A4J768 | A4J767 | A4J766 | A4J765 | A4J764 | A4J763 |
| *E. casseliflavus* | ZP_05646027.1 | ZP_05646026 | ZP 05646028 | ZP_05646029 | ZP_05646030 | ZP_05646031 | ZP_05646032.1 | ZP_05646033.1 |
| *E. gallinarum* | ZP_05650938.1 | ZP_05650937 | ZP_05650939.1 | ZP_05650940 | ZP_05650941 | ZP_05650942 | ZP_05650943.1 | ZP_05650944.1 |
| *E. coli* | P09348 | P0AF06 | P0ABW9 | P0ABX2 | P0A8T5 | P25798 | P0ABZ1 | P31068 |
| *Geobacillus sp. strain WCH70* | C5D4W8 | C5D4W9 | C5D8W1 | C5D8W2 | C5D8W3 | C5D8W4 | C5D8W5 | C5D8W6 |
| *Geobacillus sp. strain Y412MC61* | C9RXX9 | C9RXX8 | C9S078 | C9S079 | C9S080 | C9S081 | C9S082 | C9S083 |
| *H. modesticaldum* | B0TCQ0 | B0TCQ1 | B0TH99 | B0THA0 | B0THA1 | B0THA2 | B0THA3 | B0THA4 |
| *L. innocua* | Q92DW8 | Q92DW7 | Q92DX4 | Q7AP21 | Q92DX2 | Q92DX1 | Q92DX0 | Q92DT9 |
| *L. monocytogenes* | Q722L2 | Q722L1 | Q722I7 | Q722I6 | Q722I5 | Q722I4 | Q722I3 | Q722I2 |
| *L. welshimeri* | A0AGE0 | A0AGE1 | A0AGG5 | A0AGG6 | A0AGG7 | A0AGG8 | A0AGG9 | A0AGH0 |
| *Paenibacillus sp. strain JDR-2* | C6CRS7 | C6CRS6 | C6D2X6 | C6D2X5 | C6D2X4 | C6D2X3 | C6D2X2 | C6D2X1 |
| *P. aeruginosa* | Q9HXL1 | O87127/Q9HXL2 | Q9I4Q2 | Q9I4Q1 | Q51462 | Q51463 | Q51464 | A6V987 |
| *R. sphaeroides* | Q3IY87/Q3J1D0 | Q3J1D1/Q3IZ42/Q3J439/Q3IYA1 | Q3J1S6 | Q3J1S7/Q3IY73 | Q3IY74/Q3J1V8 | Q3IY91/Q3J1V7 | Q3J1V6 | Q3IY92/Q3J1V5 |
| *S. typhimurium* | P55891 | P55892 | P16437 | P0A1I7 | P26462 | P15928 | P0A1J9 | P15934 |
| *T. lettingae* | A8F892 | A8F893 | A8F3B7 | A8F3B8 | A8F3B9 | A8F8G8 | A8F8G9 | A8F8H0 |
| *T. petrophila* | A5IJB1 | A5IJB0 | A5IMK9 | A5IMK8 | A5IMK7 | A5IKK0 | A5IKK1 | A5IKK2 |
| *Thermotoga sp. strain RQ2* | B1L8D0 | B1L8C9 | B1LBW1 | B1LBW0 | B1LBV9 | B1L9T2 | B1L9T3 |  |
| *Y. enterocolitica* | A1JT92 | A1JT90 | A1JT24 | A1JT20 | A1JSX2 | A1JSX5 | A1JSX9 | A1JSV2 |

Table S6: Uniprot and NCBI accession numbers for motility proteins used for phylogenetic analysis.

|  | **FliI** | **FliJ** | **FliK** | **FlgD** | **FOP** | **FlgE** | **FlbD** | **FliL** | **FliOZ** |
| --- | --- | --- | --- | --- | --- | --- | --- | --- | --- |
| *A. tumefaciens* | O34171 |  |  | A9CK64 |  | A9CK68 |  | Q7D186 |  |
| *A. metalliredigens* | A6TRQ6 | A6TRQ5 | A6TRQ3 | A6TRQ2 | A6TRQ1 | A6TRQ0 | A6TRP9 | A6TRP6/A6TKW9 |  |
| *A. thermophilum* | B9MM22 | B9MM21 | B9MM19 | B9MM18 | B9MM17 | B9MM16 | B9MM15 | B9MM14 | B9MM10 |
| *B. amyloliquefaciens* | A7Z4P4 | A7Z4P5 | A7Z4P7 | A7Z4P8 |  |  | A7Z4Q0 | A7Z4Q1 | A7Z4Q5 |
| *B. cereus* | Q81FE3 |  |  | Q81FE2 |  | Q81FE1 |  |  |  |
| *B. thuringiensis* | Q6HKQ2 |  |  | Q6HKP9 |  | Q6HKP8 |  |  |  |
| *B. licheniformis* | Q65JM5 | Q65JM4 | Q65JM2 | Q65JM1 |  |  | Q65JL9 | Q65JL8 | Q65JL4 |
| *B. pumilus* | A8FD88 | A8FD89 | A8FD91 | A8FD92 |  |  | A8FD94 | A8FD95 | A8FD99 |
| *B. clausii* | Q5WFQ9 | Q5WFR0 | Q5WFR2 | Q5WFR3 |  |  | Q5WFR5 | Q5WFR6 | Q3V821 |
| *B. subtilis* | P23445 | P20487 | P23451 | P23455 |  |  | C0H412 | P23452 | P35536 |
| *Carnobacterium sp. AT7* | A8X5X8 | A8U5X7 | A8X5X4 | A8X5X3 | A8X5X2 | A8U5X1 | A8X5W9 | A8X5W6 | A8X5W5 |
| *C. acetobutylicum* | Q97H54 | Q97H55 | Q97H56 | Q97H57 | Q97H58 | Q97H59 | Q97H60 | Q97H61 | Q97H62 |
| *C. botulinum* | C3L1E7 | C3L1E6 | C3L1E5 | C3L1E4 | C3L1E3 | C3L1E2 | C3L1E1 | C3L1D8 | C3L1D7 |
| *C. cellulolyticum* | B8I3Q1 | B8I3Q0 | B8I3P8 | B8I3P7 | B8I3P6 | B8I3P5 | B8I3P4 |  | B8I3N9 |
| *C. kluyveri* | A5N7B7 | A5N7B8 | A5N7B9 | A5N7C0 |  | A5N7C1 | A5N7C2 | A5N7C3 | A5N7C4 |
| *C. novyi* | A0Q026 | A0Q027 | A0Q028 | A0Q029 | A0Q030 | A0Q031 | A0Q032 | A0Q035 | A0Q036 |
| *C. phytofermentans* | A9KNF8 | A9KNF7 | A9KNF5 | A9KNF4 | A9KNF3 | A9KNF2 | A9KNF1 |  | A9KNE4 |
| *C. tetani* | Q893Y3 | Q893Y4 | Q893Y5 | Q893Y6 | Q893Y7 | Q893Y8 |  | Q893Z1 | Q893Z2 |
| *D. audaxviator* | B1I5E8 | B1I5E7 | B1I5E6 | B1I5E5 | B1I5E4 | B1I5E3 |  | B1I5E2 | B1I5K3 |
| *D. acetoxidans* | C8W1G0 | C8W1G1 | C8W1G2 | C8W1G3 | C8W1G4 | C8W1G5 |  | C8W1G6 | C8W1G8 |
| *D. reducens* | A4J762 | A4J761 | A4J760 | A4J759 | A4J758 | A4J757 |  | A4J756 | A4J754 |
| *E. casseliflavus* | ZP_05646034 | ZP_05646035 | ZP_05646036.1 | ZP_05646037.1 | ZP_05646038.1 | C9AX79 | ZP_05646040.1 | ZP_05646041 | ZP_05646042.1 |
| *E. gallinarum* | ZP_05650945 | ZP_05650946 | ZP_05650947.1 | ZP_05650948.1 | ZP_05650949.1 | ZP_05650950.1 | ZP_05650951.1 | ZP_05650952 | ZP_05650953.1 |
| *E. coli* | P52612 | P52613 | P52614 | P75936 |  | P75937 |  | P0ABX8 | P52627/P22586 |
| *Geobacillus sp. strain WCH70* | C5D8W7 | C5D8W8 | C5D8X0 | C5D8X1 |  |  | C5D8X3 | C5D8X4 | C5D999 |
| *Geobacillus sp. strain Y412MC61* | C9S084 | C9S085 | C9S087 | C9S088 | D3E6T7 |  | C9S090 | C9S091 | C9S095 |
| *H. modesticaldum* | B0THA5 | B0THA6 | B0THA8 | B0THA9 | B0THB0 | B0THB1 | B0THB2 | B0THB3 | B0THB9 |
| *L. innocua* | Q92DT8 |  |  | Q92DV8 |  | Q7AP22 |  |  |  |
| *L. monocytogenes* | Q722I1 |  |  | Q722K1 |  | Q722K0 |  |  |  |
| *L. welshimeri* | A0AGH1 |  |  | A0AGF1 |  | A0AGF2 |  |  |  |
| *Paenibacillus sp. strain JDR-2* | C6D2X0 | C6D2W9 | C6D2W7 | C6D2W6 | C6D2W5 |  | C6D2W3 |  | C6D2V7 |
| *P. aeruginosa* | Q9I4N1 | Q9I4N0 | Q02JS5(PSEAB) | Q9I4Q0 |  | Q9I4P9 |  | Q9HTV7/Q9I3Q5 | Q51467 |
| *R. sphaeroides* | Q3J1V4 | Q3J1V3 | Q3J1V2 | Q3IY67/Q3J1S8 |  | Q3J1S9/Q3IYA0 |  | Q3J1V1 | Q3J1X8 |
| *S. typhimurium* | P26465 | P0A1K1 | P26416 | P0A1I9 |  | P0A1J1 |  | P26417 | P0A210/P0A1L1 |
| *T. lettingae* | A8F8H1 | A8F7A1 | A8F888 | A8F889 |  | A8F890 | A8F891 | A8F894 | A8F4W0 |
| *T. petrophila* | A5IKK3 | A5IJR1 | A5IJB5 | A5IJB4 |  | A5IJB3 | A5IJB2 | A5IJA9 | A5IJ87 |
| *Thermotoga sp. strain RQ2* | B1L9T4 | B1L8Y3 | B1L8D4 | B1L8D3 |  | B1L8D2 | B1L8D1 | B1L8C8 | B1L808 |
| *Y. enterocolitica* | A1JSV5 | A1JSV8 | A1JSW1 | A1JT17 |  | A1JT15 |  | A1JSW4 | A1JSP0/A1JSX5 |

|  | **FliP** | **FliQ** | **FlhB** | **FlhA** | **FliA** | **FlgF** | **FlgG** | **CheB** |
| --- | --- | --- | --- | --- | --- | --- | --- | --- |
| *A. tumefaciens* | Q44344 | A9CK63 | Q7D181 | Q7D165 |  | Q7D183 | Q44338 | O85128 |
| *A. metalliredigens* | A6TRP1 | A6TRP0 | A6TRN8 | A6TRN7/A6TKX3 | A6TRM8 | A6TK82 | A6TK82 | A6TRN3 |
| *A. thermophilum* | B9MM09 | B9MM08 | B9MM06 | B9MM05 | B9MLZ6 | B9MMV9 | B9MMV8 | B9MQG4/B9MMY5/B9MPE1/B9MMK8 |
| *B. amyloliquefaciens* | A7Z4Q6 | A7Z4Q7 | A7Z4Q9 | A7Z4R0 | A7Z4R8 | A7Z9L3 | A7Z4P9/A7Z9L2 | A7Z4R3 |
| *B. cereus* | Q81FC7 | Q81FC6 | Q81FC4 |  |  | Q81FC3 |  |  |
| *B. thuringiensis* | Q3V861 | Q3V860 | Q3V858 | Q6HKN6 |  | Q6HKN5 |  |  |
| *B. licheniformis* | Q65JL3 | Q65JL2 | Q65JL0 | Q65JK9 | Q65JK1 | Q65E24 | Q65JM0/Q65E25 | Q65JK6 |
| *B. pumilus* | A8FDA0 | A8FDA1 | A8FDA3 | A8FDA4 | A8FDB2 | A8FI75 | A8FD93/A8FI74 | A8FDA7 |
| *B. clausii* | Q3V822 | Q3V823 | Q3V825 | Q5WFS0 | Q5WFS6 | Q5WB89 | Q5WFR4/Q5WB90 | Q5WFS3 |
| *B. subtilis* | P35528 | P35535 | P35538 | P35620 | P10726 | P39752 | P23446/P39753 | Q05522 |
| *Carnobacterium sp. AT7* | A8X5W4 | A8X5W2 | A8X5W0 | A8X5V9 | A8X5V7 | A8X5V6 | A8X5V5 | A8X612 |
| *C. acetobutylicum* | Q97H63 | Q97H64 | Q97H65 | Q97H66 | Q97H70 | Q97H73 | Q97H74 | Q97GZ3 |
| *C. botulinum* | C3L1D6 | C3L1D5 | C3L1D4 | C3L1D3 | C3L1C9 | C3L1C6 | C3L1C5 | C3L219 |
| *C. cellulolyticum* | B8I3N8 | B8I3N7 | B8I3N5 | B8I3N4 | B8I3M5 | B8I067 | B8I066 | B8I4Z3/B8I3N1 |
| *C. kluyveri* | A5N7C5 | A5N7C6 | A5N7C7 | A5N7C8 | A5N7D1 | A5N7D3 | A5N7D4 | A5N5P6/A5MZ47 |
| *C. novyi* | A0Q037 | A0Q038 | A0Q039 | A0Q040 | A0Q044 | A0Q047 | A0Q048 | A0PZY3 |
| *C. phytofermentans* | A9KNE3 | A9KNE2 | A9KNE0 | A9KND9 | A9KNC9 | A9KRZ9 | A9KS00 | A9KND5 |
| *C. tetani* | Q893Z3 | P0CF98 | Q893Z4 | Q893Z5 | Q893Z9 | Q894A1 | Q894A2 | Q893S6 |
| *D. audaxviator* | B1I5K2 | B1I5K0 | B1I5J8 | B1I5J7 | B1I5J3 | B1I5J1 |  | B1I5H9 |
| *D. acetoxidans* | C8W1G9 | C8W1H0 | C8W1H2 | C8W1H3 | C8W1H7 | C8W1H9 | C8W1I0 | C8W0W6/C8VX95/C8W1D1/C8W1C7 |
| *D. reducens* | A4J753 | A4J752 | A4J750 | A4J749 | A4J745 | A4J744 | A4J743 | A4J796 |
| *E. casseliflavus* | ZP_05646043.1 | ZP_05646044 | ZP_05646046.1 | ZP_05646047 | ZP_05646048 | ZP_05646049.1 | ZP_05646050.1 | ZP_05646055.1 |
| *E. gallinarum* | ZP_05650954.1 | ZP_05650955 | ZP_05650957.1 | ZP_05650958 | ZP_05650959 | ZP_05650960.1 | ZP_05650961.1 | ZP_05650966.1 |
| *E. coli* | P0AC05 | P0AC07 | P76299 | P76298 | P0AEM6 | P75938 | P0ABX5 | P07330 |
| *Geobacillus sp. strain WCH70* | C5D9A0 | C5D9A1 | C5D9A3 | C5D9A4 | C5D9B2 | C5D968 | C5D8X2/C5D967 | C5D9A7 |
| *Geobacillus sp. strain Y412MC61* | C9S096 | C9S097 | C9S099 | C9S0A0 | C9S0A8 | C9RWK7 | C9S089/C9RWK6 | C9S0A3 |
| *H. modesticaldum* | B0THC0 | B0THC1 | B0THC3 | B0THC4 | B0THD2 | B0THM0 | B0THL9 | B0TG88/B0THC7 |
| *L. innocua* | Q92DX7 | Q92DX6 | Q92DX4 | Q92DX3 |  | Q92DX1 |  |  |
| *L. monocytogenes* | Q722L8 | Q3V885 | Q3V883 | Q722L7 |  | Q722L5 |  |  |
| *L. welshimeri* | A0AGD1 | A0AGD2 | A0AGD4 | A0AGD5 |  | A0AGD7 |  |  |
| *Paenibacillus sp. strain JDR-2* | C6D2V6 | C6D2V5 | C6D2V3 | C6D2V2 | C6D2X4 | C6D5C1 | C6D2W4/C6D5C0 | C6D2X9 |
| *P. aeruginosa* | Q51468 | Q9I3Q4 | Q9I3Q2 | Q9I3P9 | P29248 | Q9I4P8 | Q9I4P7 | Q9I6V9/Q9HXT8/O87125 |
| *R. sphaeroides* | Q3J1X7/Q3IY94 | Q3J1X6/Q3IY75 | Q3IY82/Q3J1X4 | Q3IY84/Q3J1Y1 | Q3J1Y3 | Q3J1T0 | Q3J1T1 | Q3J653/Q3J1W3 |
| *S. typhimurium* | P54700 | P0A1L5 | P40727 | P40729 | P0A2E8 | P16323 | P0A1J3 | P04042 |
| *T. lettingae* | A8F4V9 | A8F4V8 | A8F4V6 | A8F4V5 | A8F4X9 | A8F457 | A8F458 | A8F579 |
| *T. petrophila* | A5IJ88 | A5IJ89 | A5IIM4 | A5IIM5 | A5IIN1 | A5IM40 | A5IM41 | A5IK11 |
| *Thermotoga sp. strain RQ2* | B1L809 | B1L810 | B1LC25 | B1LC26 | B1LC32 | B1LB52 | B1LB51 | B1L982 |
| *Y. enterocolitica* | A1JSX7 | A1JSY2 | A1JT48 | A1JT43 | A1JSP4 | A1JT11 | A1JT09 | A1JT63 |

|  | **CheR** | **CheA** | **CheY** | **FliM** | **FliNY** |
| --- | --- | --- | --- | --- | --- |
| *A. tumefaciens* | Q7D1A6 | A9CK76 | Q7D1A5/Q7D1A8 | Q44457 | Q57259 |
| *A. metalliredigens* | A6TRF7 | A6TRN2 | A6TRP3/A6TKX2 | A6TRP5/A6TKX0 | A6TKX1 |
| *A. thermophilum* | B9MQJ4/B9MPE4/B9MMK4 | B9MM01/B9MMK3/B9MPE2/B9MKK5 | B9MM11 | B9MM13 | B9MM12 |
| *B. amyloliquefaciens* | A7Z624 | A7Z4R4 | A7Z4Q4 | A7Z4Q2 | A7Z4Q3 |
| *B. cereus* | Q81FF8/Q81H19 | Q7BYD5 | Q81FG3 | Q81FD0 | Q81FD1/Q81FC9 |
| *B. thuringiensis* | Q6HKR7/Q6HMG6 | Q6HKS1 | Q6HKS2 | Q6HKN9 | Q6HKP0/Q6HKN8 |
| *B. licheniformis* | Q65I27 | Q65JK5 | Q65JL5 | Q65JL7 | Q65JL6 |
| *B. pumilus* | A8FEK6 | A8FDA8 | A8FD98 | A8FD96 | A8FD97 |
| *B. clausii* | Q5WGS9 | Q5WEX3 | Q5WFR9 | Q5WFR7 | Q5WFR8 |
| *B. subtilis* | P31105 | P29072 | P24072 | P23453 | P24073 |
| *Carnobacterium sp. AT7* | A8U610 |  | A8U606 | A8X604 |  |
| *C. acetobutylicum* | Q97GZ4/Q97MS0 | Q97GZ5/Q97MS3 | Q97GZ7 | Q97GZ9 | Q97H00 |
| *C. botulinum* | C3L218 | C3L217 | C3L215 | C3L213 | C3L212 |
| *C. cellulolyticum* | B8I2T5 | B8I3N0/B8I4Z7 | B8I3P0 | B8I3P2 | B8I3P1 |
| *C. kluyveri* | A5MZ46 | A5MZ45/A5N784 | A5N783 | A5MZ41 | A5MZ40 |
| *C. novyi* | A0PZY4 | A0PZY5 | A0PZY7 | A0PZY9 | A0PZZ0 |
| *C. phytofermentans* | A9KLR5 | A9KND4 | A9KNE5 | A9KNE7 | A9KNE6 |
| *C. tetani* | Q893S7 | Q893S8 | Q893T0 | Q893T2 | Q893T3 |
| *D. audaxviator* | B1I5I9 | B1I5I0 | B1I5I7 | B1I5G5 | B1I5G4/B1I5E1 |
| *D. acetoxidans* | C8W1I2/C8W3H5/C8VX96/C8W0W5 | C8W1C6/C8W0W2 | C8W1I4 | C8W1I5 | C8W1I6 |
| *D. reducens* | A4J741/A4J3Z9 | A4J797 | A4J739 | A4J738 |  |
| *E. casseliflavus* | ZP_05646056.1 | ZP_05646057.1 | ZP_05646059 | C9AXA1 | ZP_05646062.1 |
| *E. gallinarum* | ZP_05650967.1 | ZP_05650968.1 | ZP_05650970 | C9A3Y8 | ZP_05650973.1 |
| *E. coli* | P07364 | P07363 | P0AE67 | P06974 | P15070 |
| *Geobacillus sp. strain WCH70* | C5D3E2 | C5D9A8 | C5D998 | C5D996 | C5D997 |
| *Geobacillus sp. strain Y412MC61* | C9S068 | C9S0A4 | C9S094 | C9S092 | C9S093 |
| *H. modesticaldum* | B0TAG3 | B0THC8/B0TBE2/B0TG87/B0TC94 | B0THB8 | B0THB4 | B0THB7 |
| *L. innocua* | Q92DX0 | Q92DW2 | P0A4H6 | Q92DV5 | Q92DV6/Q92DV4/Q92DW1 |
| *L. monocytogenes* | Q722L4 | Q722K5 | Q722K6 | Q722J8 | Q722J9/Q722J7/Q722K4 |
| *L. welshimeri* | A0AGD8 | A0AGE7 | A0AGE6 | A0AGF4 | A0AGF3/A0AGF5/A0AGE8 |
| *Paenibacillus sp. strain JDR-2* | C6CUC8/C6D7E2/C6CXS4 | C6D2X8 | C6D2V8 | C6D2W0 | C6D2V9 |
| *P. aeruginosa* | O87131/Q9I6V7 | O87124 | Q51455 | Q51465 | Q51466 |
| *R. sphaeroides* | Q3J1W2/Q3J654/Q3J3N9 | Q3J1W8/Q3J3N7/Q3J657 | Q3J3P0/Q3J3N6/Q3J3N0/Q3IX24/Q3J658/Q3J1W7 | Q3J1V0/Q3J1J3 | Q3J1X9 |
| *S. typhimurium* | P07801 | P09384 | P0A2D5 | P26418 | P26419 |
| *T. lettingae* | A8F5Z5 | A8F4W3 | A8F4W1 | A8F895 | A8F896 |
| *T. petrophila* | A5IJV7 | A5IJ84 | A5IJ86 | A5IJA8 | A5IJA7 |
| *Thermotoga sp. strain RQ2* | B1L929 | B1L805 | B1L807 | B1L8C7 | B1L8C6 |
| *Y. enterocolitica* | A1JT66 | A1JT85 | A1JT60 | A1JSW7 | A1JSX0 |

|  | **FlgM** | **FlgN** | **FlgK** | **FlgL** | **FliC** | **FlaG** | **FliD** | **FliS** |
| --- | --- | --- | --- | --- | --- | --- | --- | --- |
| *A. tumefaciens* |  |  | A9CK67 | A9CK66 | Q7D189/Q7D179/ |  |  |  |
| *A. metalliredigens* | A6TL82 | A6TL83 | A6TL84 | A6TL85 | A6TLF2/A6TL89 | A6TLE1/A6TP26 | A6TLE2 | A6TLE3 |
| *A. thermophilum* | B9MKB3 | B9MKB2 | B9MKB1 | B9MKB0 | B9MKA0 | B9MK90 | B9MK89 | B9MK88 |
| *B. amyloliquefaciens* | A7Z9B2 | A7Z9B1 | A7Z9B0 | A7Z9A9 | A7Z9A5 |  | A7Z9A4 | A7Z9A3 |
| *B. cereus* |  |  | Q81FF4 | Q81FF3 | Q81FD3/Q81FD4/Q81FD5/Q81FD6 |  | Q81FF2 | Q81FF1 |
| *B. thuringiensis* |  |  | Q6HKR3 | Q6HKR2 | Q6HKP2/Q6HKP3 |  | Q6HKR1 | Q6HKR0 |
| *B. licheniformis* | Q65EB1 | Q65EB2 | Q65EB3 | Q65EB4 | Q65EB8 | Q65EB9 | Q65EC0 | Q65EC1 |
| *B. pumilus* | A8FHX9 | A8FHX8 | A8FHX7 | A8FHX6 | A8FC67/A8FC66/A8FC68/A8FC69/A8F9D2 | A8FHX2 | A8FHX1 | A8FHX0 |
| *B. clausii* | Q5WDE5 | Q5WDE6 | Q5WDE7 | Q5WDE8 | Q5WBM8 | Q5WDF1 | Q5WDF2 | Q5WDF3 |
| *B. subtilis* | P39809 | P39808 | P39810 | P96501 | P02968 | P39737 | P39738 | P39739 |
| *Carnobacterium sp. AT7* | A8X601 | A8X600 | ZP_02184101 | A8X5Z6 | A8U5Z1 | A8X5Z0 | A8X5Y9 | A8X5Y6 |
| *C. acetobutylicum* | Q97H01 | Q97H02/Q97H02 | Q97H03 | Q97H04 | Q97IK6/Q97H04/Q97IT5/Q97H46/O69136 | Q97H07 | Q97H10 | Q97H09 |
| *C. botulinum* | C3L211 | C3L210/C3L210 | C3L209 | C3L208 | C3L1W8/C3L1U0/C3KZ58/C3L200/C3L1Z9 | C3L205 | C3L202 | C3L203 |
| *C. cellulolyticum* | B8I4C6 | B8I4C7/B8I4E1 | B8I4C8/B8I4C9 | B8I4D0 | B8I4D4/B8I4D5/B8I4D6 | B8I4D8 | B8I4D9 | B8I4E0 |
| *C. kluyveri* | A5MZ39 | A5MZ38/A5MZ38 | A5MZ37 | A5MZ35 | A5MZ27 | A5MZ32 | A5MZ29 | A5MZ30 |
| *C. novyi* | A0PZZ1 | A0PZZ2 | A0PZZ3 | A0PZZ4 | A0Q018/A0Q019/A0Q003/A0Q002 | A0PZZ7 | A0Q000 | A0PZZ9 |
| *C. phytofermentans* | A9KSP6 |  | A9KSP9/A9KSP8 | A9KSQ0 | A9KL48/A9KSQ8 | A9KSQ4 | A9KSQ5 | A9KSQ6 |
| *C. tetani* | NT02CT1841 | Q893T4/Q893T4 | Q893T5 | Q893T6 | Q893W6/Q893X7/Q893U2 | Q893T8 | Q893X0 | Q893T9 |
| *D. audaxviator* | B1I5H8 | B1I5H7/B1I5H7 | B1I5H6 | B1I5H5 | B1I5G9 | B1I5H0 | B1I5H1 | B1I5H2 |
| *D. acetoxidans* | C8W1E4 | C8W1E3/C8W1E3 | C8W1E2 | C8W1E1 | C8W1D2 | C8W1D8 | C8W1D6 | C8W1D7 |
| *D. reducens* | A4J795 |  | A4J793 | A4J792 | A4J786/A4J790 | A4J770 | A4J775 | A4J772 |
| *E. casseliflavus* | ZP_05646063 | ZP_05646064.1 | ZP_05646065 |  | EEV29400 | ZP_05646068.1 | ZP_05646069 | ZP_05646071.1 |
| *E. gallinarum* | ZP_05650974 | ZP_05650975.1 | ZP_05650976 |  | ZP_05650978 | ZP_05650979.1 | ZP_05650980 | ZP_05650982.1 |
| *E. coli* | P0AEM4 | P43533 | P33235 | P29744 | P04949 |  | P24216 | P26608 |
| *Geobacillus sp. strain WCH70* | C5D7X0 | C5D7T9 | C5D7T8 | C5D7T7 | C5D7T2/C5D7U3 | C5D7S | C5D7S5 | C5D7S4/C5D866 |
| *Geobacillus sp. strain Y412MC61* | C9RVR9 | C9RVR8 | C9RVR7 | C9RVR6 | C9RVE6/C9RVS2 | C9RVC6 | C9RVC5 | C9RVC4/C9RVS3 |
| *H. modesticaldum* | B0TH48 | B0TH47 | B0TH44 | B0TH43 | B0TH01 | B0TH34 | B0TH38 | B0TH36/B0TH00 |
| *L. innocua* |  |  | Q92DX9 | Q92DX8 | Q92DW3 |  | Q92DX7 | Q92DX6 |
| *L. monocytogenes* |  |  | Q722J2 | Q722J1 | Q722K7 |  | Q722J0 | Q722I9 |
| *L. welshimeri* |  |  | A0AGG0 | A0AGG1 | A0AGE5 |  | A0AGG2 | A0AGG3 |
| *Paenibacillus sp. strain JDR-2* | C6D595 | C6D594 | C6D593 | C6D592 | C6D588 | C6D4W0 | C6D4V9 | C6D4V8 |
| *P. aeruginosa* | Q9HYP5 |  | Q9I4P3 | Q9I4P2 | P72151 | O33420/A3L9X7/P72152/Q9I4N7 | Q9K3C5 | Q9I4N6 |
| *R. sphaeroides* | Q3J1X7 |  | Q3J1T5 | Q3J1T6 | Q3J1U0 |  | Q3J1T9 | Q3J1X0 |
| *S. typhimurium* | P26477 | P0A1J7 | P0A1J5 | P16326 | P52616/P06179 |  | P16328 | P26609 |
| *T. lettingae* | A8F506 |  | A8F504 | A8F503 | A8F3U3 | A8F3K8 | A8F3K7 | A8F8R4 |
| *T. petrophila* | A5IKY5 |  | A5IKY7 | A5IKY8 | A5IJ26 | A5IN55 | A5IN56 | A5IN07 |
| *Thermotoga sp. strain RQ2* | B1LA66 |  | B1LA68 | B1LA69 | Q4FF54 | Q4FF29 | B1LCW8 | B1LCR6 |
| *Y. enterocolitica* | A1JT31 | A1JT33 | A1JSZ7 | A1JSZ5 | A1JSQ5/A1JSQ9/A1JSQ1 |  | A1JSR2 | A1JSR5 |
